# Age- and Sex-Related Cortical Gray Matter Volume Differences in Adolescent Cannabis Users: A Systematic Review and Meta-Analysis of Voxel-Based Morphometry Studies

**DOI:** 10.3389/fpsyt.2021.745193

**Published:** 2021-12-01

**Authors:** Aliyah Allick, Grace Park, Kwon Kim, Michelle Vintimilla, Krutika Rathod, Rachael Lebo, Julie Nanavati, Christopher J. Hammond

**Affiliations:** ^1^Division of Child and Adolescent Psychiatry, Johns Hopkins University School of Medicine, Baltimore, MD, United States; ^2^Department of Psychiatry and Behavioral Sciences, Johns Hopkins University School of Medicine, Baltimore, MD, United States; ^3^Welch Medical Library, Johns Hopkins University School of Medicine, Baltimore, MD, United States; ^4^I.D. Weeks Library, Health Sciences Department, University of South Dakota, Vermillion, SD, United States; ^5^Behavioral Pharmacology Research Unit, Johns Hopkins University School of Medicine, Baltimore, MD, United States

**Keywords:** adolescence, cannabis use and dependence, development, age, brain structural alterations, voxel-based morphometry, sex

## Abstract

**Introduction:** Adolescent-onset cannabis use is rising in the era of marijuana legalization. Recent imaging studies have identified neuroanatomical differences between adult cannabis users and controls that are more prominent in early-onset users. Other studies point to sex-dependent effects of cannabis.

**Methods:** A systematic review following PRISMA guidelines and subsequent effect-size seed-based d mapping (SDM) meta-analyses were conducted to investigate relationships between age (across the 12-to-21-year-old developmental window), sex, and gray matter volume (GMV) differences between cannabis using (CU) and typically developing (TD) youth.

**Results:** Our search identified 1,326 citations, 24 of which were included in a qualitative analysis. A total of 6 whole-brain voxel-based morphometry (VBM) studies comparing regional GMV between 357 CU [mean (SD) age = 16.68 (1.28); 71% male] and 404 TD [mean (SD) age = 16.77 (1.36); 63% male] youth were included in the SDM-meta-analysis. Meta-analysis of whole-brain VBM studies identified no regions showing significant GMV difference between CU and TD youth. Meta-regressions showed divergent effects of age and sex on cortical GMV differences in CU vs. TD youth. Age effects were seen in the superior temporal gyrus (STG), with older-aged CU youth showing decreased and younger-aged CU youth showing increased STG GMV compared to age-matched TD youth. Parallel findings in the STG were also observed in relation to duration of CU (years) in supplemental meta-regressions. Regarding sex effects, a higher proportion of females in studies was associated with increased GMV in the middle occipital gyrus in CU vs. TD youth.

**Conclusions:** These findings suggest that GMV differences between CU and TD youth, if present, are subtle, and may vary as a function of age, cumulative cannabis exposure, and sex in young people. Whether age- and sex-related GMV differences are attributable to common predispositional factors, cannabis-induced neuroadaptive changes, or both warrant further investigation.

## Introduction

Cannabis is the most commonly used federally illicit, psychoactive drug by U.S. adolescents and young adults, and the most common drug problem that teens receive substance use treatment for in the U.S. (1, 2). Over 1.6 million adolescents between the ages of 12 and 17 and 7.6 million young adults between the ages of 18 and 25 residing in the U.S. report current use of cannabis (1). In 2018, 24% of U.S. high school seniors reported past-30-day use of cannabis with 6.4% reporting daily use (3, 4). Patterns of cannabis use (CU) have changed among U.S. youth during the era of cannabis legalization. Over the past two decades, legalization of cannabis for medical and recreational use by a majority of U.S. states has dramatically altered societal perceptions of youth and their parents, resulting in a more permissive environment and increased access to cannabis, including new cannabis products (e.g., concentrates, edibles, vaped cannabis) with high concentrations of delta-9-tetrahydrocannabinol (Δ-9-THC), the main psychoactive component of cannabis (5). While population-wide use of cannabis by adolescents has not changed appreciably in the past 10 years, recent studies point to increased prevalence of daily CU and expanded use of concentrates and vaped cannabis among U.S. youth, along with increased prevalence of CU among different subgroups (e.g., college-aged young adults) (5). This is problematic given growing literature that recreational use of cannabis, particularly high-Δ-9-THC-potency cannabis, during adolescence is associated with numerous adverse health outcomes including increased risk for psychiatric disorders, academic failure, and higher rates of morbidity and mortality (6, 7).

The use of cannabis during adolescence may have complex effects on brain structure and function that extend into adulthood (8). While preclinical studies show strong and consistent evidence for a causal relationship between exposure to cannabinoids and changes in brain morphology [see (9) and (10) for reviews], there is conflicting evidence on the long-term effects of cannabis on brain structure in humans (11). Evidence from human structural magnetic resonance imaging (sMRI) studies has been mixed to date, with some studies reporting increased brain volumes related to CU (12) and other studies reporting decreased brain volumes (13) or the absence of volumetric differences between CU and non-users (11). Factors thought to contribute to the variability in human sMRI findings related to cannabis exposure include age of cannabis initiation and onset of regular use, frequency and chronicity of use, co-occurrence/comorbidity of CU with other substance use and substance use disorders such as alcohol and tobacco, and the presence of comorbid psychiatric disorders (11, 14). Many of these confounding factors also emerge during adolescence, a period of increased sensitivity to the negative effects of Δ-9-THC, alcohol, and nicotine exposure (8).

One understudied factor that may account for some of the variance observed in morphologic findings is the age or developmental period at which cannabis exposure effects are investigated. Based upon systematic examination of the adult sMRI literature [see (15) for review], adult studies typically show evidence of decreased gray matter volume (GMV) between CU adults and age-matched non-using controls. Compared to age-matched controls, decreased GMV in CU adults [especially heavy users (15, 16), dependent users (17, 18), and those who initiated cannabis before age 16 (19, 20)] has been observed across diverse brain regions with elevated cannabinoid receptor type 1 (CB1) expression including the medial temporal cortex, temporal pole, hippocampus/parahippocampal gyrus, insula, amygdala, thalamus, prefrontal cortex (PFC), orbitofrontal cortex (OFC), and cerebellum. Relatedly, a recently published meta-analysis of adult sMRI studies showed that regular CU adults had decreased hippocampal and medial and lateral OFC volumes compared to age-matched controls (21). Other adult sMRI studies have shown no neuroanatomical differences between CU adults and age-matched controls (11, 22). Notably absent from this literature are GMV studies in adults that show increased cortical thickness or GMV in relation to cannabis use (21), although one or two studies have reported increased volumes in non-cortical regions including the striatum (23) and cerebellum (15). In contrast, based upon systematic examination of the adolescent sMRI literature [see (24) for review], more variability in morphologic findings is seen, and the opposite pattern of cannabis-related GMV abnormalities is observed, with a number of studies showing larger GMV volumes in CU compared to typically-developing (TD) youth (12, 25, 26). Across these studies, differences in GMV between CU and TD youth are primarily seen in the same brain regions as those observed in CU adults (e.g., amygdala, hippocampus, PFC, cerebellum). Using data from the IMAGEN trial, Orr et al. (12) found evidence for increased GMV in the amygdala, hippocampus, striatum, left PFC, lingual gyrus, posterior cingulate, and cerebellum in a sample of 14-year-old low-level CU compared to age-matched TD youth. Another study by Medina et al. (26) reported increased hippocampal volumes in adolescent CU compared to TD youth. Not all studies have shown increased GMV in adolescent CU compared to age-matched youth. In addition to studies showing null findings (11), some studies have conversely shown decreased GMV in CU vs. age-matched TD youth (27), although these have been primarily in late adolescent or young adult samples. When taken together, the collective findings across adult and adolescent sMRI studies suggest the possibility of an age/developmental gradient with regard to the effects of cannabis exposure on cortical morphology. As such, age-related influences on the relationship between CU and morphology warrant further investigation, especially across adolescence and young adulthood (ages 12-21 years), the main time period of peak cannabis exposure and cortical maturational changes.

Another factor that could account for variance in morphological findings across studies is the distribution of females-to-male participants in studies. There is growing evidence in support of sex differences in the development, clinical and behavioral presentation, and neural correlates of CU from both clinical and preclinical studies (28). Women begin using cannabis at a later age than men and progress more quickly from first use to dependence (known as the “telescoping” effect) (29), although this pattern is less pronounced in adolescents. Women also report greater abuse-related subjective effects, withdrawal severity, and cannabis-related problems, along with higher rates of comorbid mood and anxiety disorders compared to men (28, 30, 31). In preclinical studies, female rodents show greater sensitivity to the anxiogenic, reinforcing, and sedative effects of cannabinoids (32). While preclinical adolescent cannabis exposure studies largely show widespread desensitization and downregulation of CB1 receptors in the brains of both male and female rodents, some studies also point to sex-specific effects in the cerebellum, hippocampus, PFC, amygdala, and striatum (28, 33). Recent human imaging studies indicate that sex may moderate the relationship between CU and brain morphometry in PFC, ACC, cerebellar, and amygdala regions in adolescents and adults (34, 35). Results from two studies in CU adolescents found that female cannabis users had increased PFC and amygdala volumes compared to female controls, while male cannabis users had smaller volumes or no volumetric differences from male controls (26, 34) [conversely see (36)]. These findings indicate the need for future imaging studies to determine how sex influences the neuroanatomical alterations observed in relation to cannabis exposure in humans.

Given the changing legal status of cannabis and potential for negative downstream effects on health indices for American youth, it is increasingly important to understand the effects of CU on neurodevelopment. Major time sensitive goals of the scientific field today are to determine if neuroanatomical abnormalities emerge as a result of adolescent cannabis exposure, and if present, whether these abnormalities mediate the relationship between cannabis exposure during adolescence and adverse health outcomes in adulthood. Variability in morphological findings across studies in the nascent literature warrant further investigation, especially, to determine whether some of the variance across studies is the result of age/developmental effects or cumulative cannabis exposure, and whether sex-dependent effects are present. Obtaining a comprehensive understanding of neurodevelopmental and sex-dependent effects of CU on GMV requires meta-analysis of sMRI studies examining adolescent boys and girls at various developmental stages. As such, the present study, a whole-brain voxel-based morphometry (VBM) meta-analysis, focused on age-related and sex-related cortical and subcortical GMV differences in relation to CU across adolescence and young adulthood. Using effect-size seed-based d mapping (SDM, also known as signed differential mapping) (37), a coordinate-based meta-analytic approach on whole-brain VBM studies comparing CU and TD youth, our study aims were three-fold: (1) to identify brain regions of increased or decreased GMV in CU relative to TD youth, (2) to explore whether specific regional GMV differences in CU vs. TD youth are age-related (i.e., do they vary as a function of age), and (3) to determine if regional GMV differences in CU vs. TD youth are sex-dependent (i.e., do they vary as a function of the distribution of females-to-male participants in the sample). Based upon previous VBM studies (12, 34), we hypothesized that CU and TD youth would show GMV differences in brain regions with elevated CB1 receptor expression including the medial temporal lobe, hippocampus, amygdala, PFC, OFC, and cerebellum, and that these GMV differences would vary as a function of age and sex. Specifically, we predicted that increasing age across adolescence would be associated with decreasing GMV in these brain regions in CU youth compared to age-matched TD youth and that increasing proportion of female participants in studies would be associated with increasing GMV in these regions in CU youth compared to sex-matched TD youth.

## Materials and Methods

A systematic review of peer-reviewed studies was conducted following the Preferred Reporting Items for Systematic Reviews and Meta-analyses (PRISMA) guidelines and methods (38). A subset of the studies from the review that included coordinate-level data or parametric maps were used in the SDM meta-analyses.

### Search Strategy

We searched for studies indexed in the online databases PubMed/Medline, Cochrane, Embase, and Web Science from January 1990 to November 2019 using the following search terms: “Adolescent”[Mesh] OR “adolescent” OR “young adult” OR “youth” OR “teenager” AND “Neuroimaging”[Mesh] OR “Magnetic Resonance Imaging”[Mesh] OR “MRI” OR “structural MRI” OR “sMRI” OR “voxel-based morphometry” OR “VBM” OR “voxel-based” OR “voxel-wise” OR “neuroimaging” OR “brain circuit” OR “neural” AND “Cannabis-Related Disorders”[Mesh] OR “cannabis use” OR “marijuana use” OR “cannabis abuse” OR “marijuana abuse” OR “cannabis dependenc^*^[tiab]” OR “marijuana dependenc^*^[tiab]” OR “cannabis addiction” OR “marijuana addiction” OR “cannabis use disorder” OR “marijuana use disorder” OR “cannabis^*^[tiab]” OR “marijuana^*^[tiab]” OR “marihuana^*^[tiab]” OR “Δ-9-tetra-hydrocannabidol” OR “THC”. Broad search terms were used to minimize the likelihood of the search not identifying all relevant studies. In addition, we manually scanned the references of included studies and cross-referenced relevant original research, reviews, and meta-analyses to identify studies that may have been missed by the search.

### Study Selection

Studies were selected if they met the following criteria: (1) included > 10 participants; (2) participants were between the ages of 12 and 21 years; (3) used diagnostic criteria for cannabis use disorder (CUD) as specified by the DSM (DSM-IV or DSM-5) or described frequency of cannabis use (e.g., daily, weekly, etc.) in study participants; (4) used whole-brain VBM and voxel-wise analyses; (5) reported within- or between-subject contrasts in GMV across cannabis use (CU) and typically developing (TD) control youth, or brain-behavior correlations between GMV and cannabis-related variables; (6) reported coordinates from the above whole-brain analyses in standardized anatomic space [i.e., Talairach or Montreal Neurologic Institute (MNI) space] and (7) provided information about the inclusion/exclusion criteria, clinical characteristics, and demographics of the study sample.

Articles that studied adolescent CU within the context of co-occurring psychiatric disorders were included if studies also included controls that did not use cannabis. Studies with young adult samples were included if they the mean age of participants was below 21 years.

### Data Extraction

Articles were extracted, organized, and reviewed using Covidence software (covidence.org). Initial independent title and abstract evaluations were done to identify potential articles of interest by two authors (A.A. and K.R.). Data extraction accuracy showed high correspondence/agreement (>80%) between reviewers. Abstract evaluation was followed by an independent full-text review of articles. Group discussion was used to resolve uncertainties about inclusion criteria and finalize the list of articles included in the qualitative review and SDM meta-analysis.

To facilitate exploration and interpretation of results, studies that examined GMV differences but failed inclusion criteria due to lack of statistical maps or whole-brain analytic approaches were retained for the purposes of qualitative analysis.

To create the final list of studies included in the meta-analysis, we took a three-step approach: Studies identified with the above search that reported coordinates of anatomical differences in CU groups from whole-brain analyses in Talairach or MNI space were identified and marked for inclusion in the SDM meta-analysis. For those studies and for whole-brain VBM studies that provided insufficient information on coordinates, corresponding authors were contacted via email to determine if unthresholded statistical maps or coordinates could be provided. Additionally, we searched NeuroVault (neurovault.org) using select search terms (from above) to try to find unthresholded statistical maps from the relevant studies. These approaches did not yield additional studies or unthresholded statistical maps. Thus, peak coordinates from published data were used for the meta-analysis.

### Data Analysis

#### SDM Meta-Analysis Procedures

All meta-analyses were carried out using the anisotropic effect-size signed differential mapping permuting subject images (SDM-PSI) software, v.6.21 (http://www.sdmproject.com). SDM-meta-analysis is a statistical technique for meta-analyzing neuroimaging data that approach that recreates voxel-level maps of effect sizes and their variance based upon T-maps (37). In contrast to other meta-analytic approaches, SDM enables original statistical parametric maps and peak coordinates to be combined, and reconstructs positive and negative effects within the same statistical maps, preventing a voxel from appearing in opposite directions, and providing for more accurate representation of the results.

#### Data Coding and Preparation for SDM Meta-Analysis

In preparation for the SDM meta-analysis, the following data coding steps were taken: For studies that met inclusion criteria, coordinates associated with CU groups or variables were manually recorded by two authors (A.A. and C.J.H.). Coded anatomical foci were then double screened for accuracy. If the studies reported coordinates in either Talairach or MNI coordinates, a text file containing the reported coordinates and the t-score associated with those coordinates was created. If a study reported multiple experiments, the results were still reported in the same text file. *P*-values or *z*-values were converted into t-scores using SDM Utilities calculator, otherwise sign of their effect was reported as positive or negative. In addition, a table was made the study identifier (main author), the t-score used to determine significance, and the number of people in the experimental and control groups. If a study reported a statistically significant corrected *p*-value, but didn't give provide sufficient information to transform the corrected *p*-value into a t-score, a t-score of 3.1 was used, providing a conservative estimate. Studies that had no significant peaks were also included. To prepare for the meta-regressions, data on CU and TD youth's age at time of scan, proportion of female participants, age range, average days of cannabis use in past-30-days, and duration of cannabis use (years) were obtained for each study and included as variables.

#### Meta-Analysis Procedures

The main analysis was conducted in two steps: First SDM meta-analyses were conducted on the statistical parametric maps showing group-level effects for each study to examine for unadjusted differences between youth with CU and matched TD youth. Next, two linear meta-regressions were conducted, one using mean age (years) at time of scan and the other using the proportion of females to males from each study as dependent variables to examine effects of increasing age across adolescence and increasing proportion of female sex on GMV. All models were thresholded using an uncorrected *p*-value < 0.005 consistent with other SDM meta-analyses (37). Familywise error correction was also carried out using 1,000 permutations, then thresholded using a corrected *p*-value of 0.05.

#### Reliability Analysis and Supplemental Subgroup Meta-Analyses and Meta-Regressions

To establish the reliability of our meta-analytic results, a jackknife analysis was performed by removing a single dataset and repeating the analysis in sequence. This was done for the primary SDM meta-analysis and meta-regression analyses. Supplemental subgroup meta-analyses were conducted to examine subgroup effects in (1) studies that controlled for alcohol and tobacco use, (2) studies that excluded youth with comorbid psychiatric disorders, and (3) studies with samples restricted to youth who met CUD diagnostic criteria. Supplemental linear meta-regression analyses were used to examine the influence of (1) age range, (2) mean days of CU in the past 30 days (indexing recent CU), and (3) mean years of cannabis use (indexing duration of CU) on GMV differences between CU and TD youth.

## Results

### Systematic Review and Qualitative Analysis

**Figure 1:**
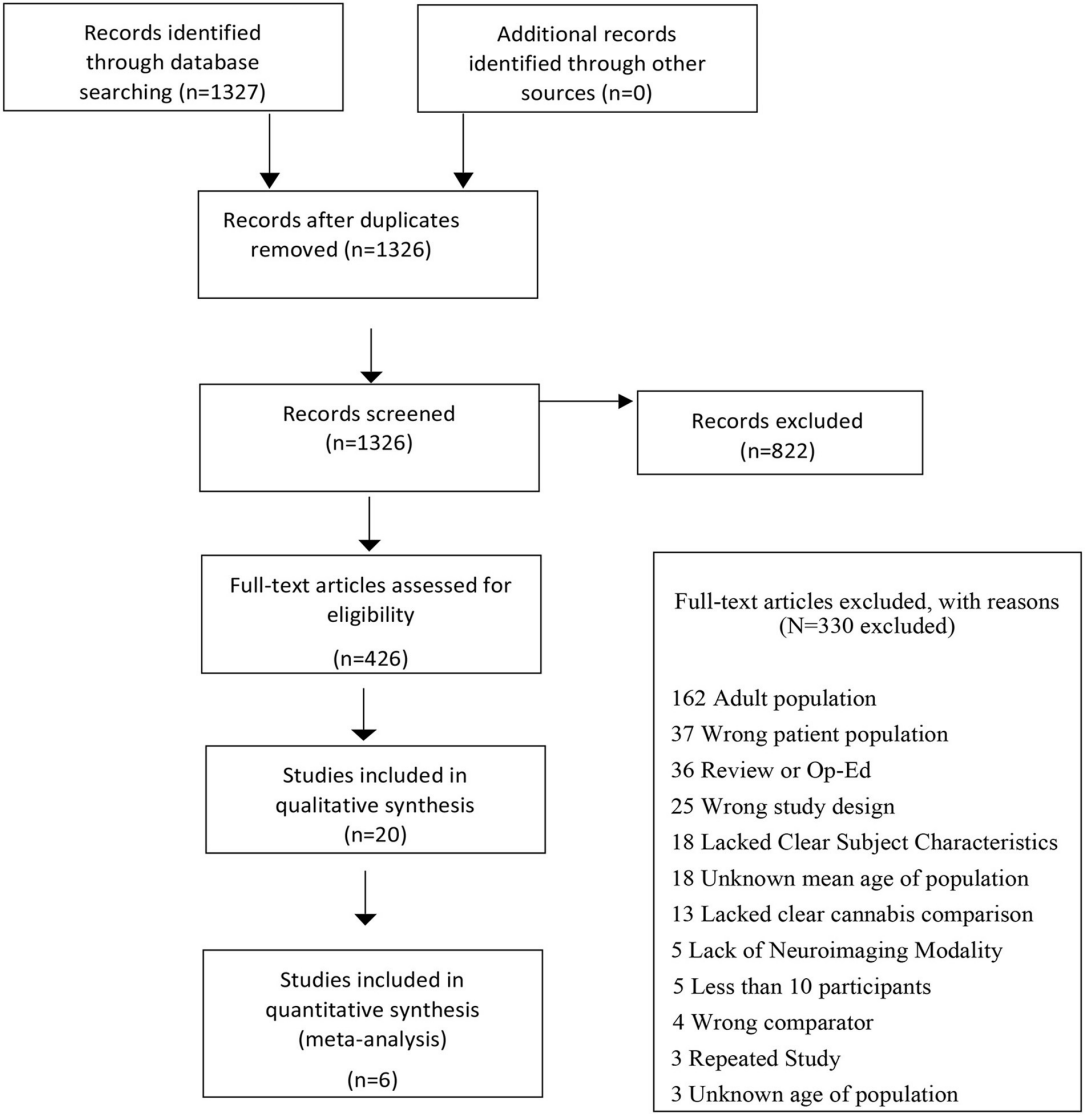
Flowchart outlining selection procedure of studies of GMV differences.

The initial search identified 1,327 citations with 822 records excluded following title and abstract screen. Out of 436 citations that underwent full text review, 20 studies examining GMV differences were included in the qualitative analysis, 6 of which met all inclusion criteria. A PRISMA flow diagram depicting the search process is presented in Figure 1 and results from the qualitative analysis (Supplementary Results S1 and Supplementary Table S1) are presented in the supplement.

### Study and Sample Characteristics

Six eligible whole-brain VBM studies (11, 12, 39–42) that involved a direct comparison of GMV between CU youth [*n* = 357; mean (SD) age = 16.68 (1.28); age range 14-25 years] and TD youth [*n* = 403; mean (SD) age = 16.77 (1.36); age range 14-25 years] were included in the SDM meta-analysis (Table 1). One hundred and five (29.4%) of the 357 CU youth and 151 (37.5%) of the 403 TD youth from the six eligible studies were female. In the meta-analytic sample, the mean ages (t = 0.01, *p* = *0.99*) and proportion of participants who were of female sex (t = −0.05, *p* = *0.96*) did not significantly differ across CU and TD groups. One study (42) examined GMV differences between CUD and non-CUD participants with bipolar disorder (BP). All analyses were run with and without this study. Four of six studies controlled for alcohol use in their main analyses and five of six controlled for tobacco use (Supplementary Table S2).

**Table 1:** Summary of VBM studies included in the meta-analysis.

**References**	**Diagnosis**	**No. of CU youth** **(% male)**	**Age of CU youth, mean (range)**	**Quantity of CU by CU** **youth**	**No. of TD youth** **(% male)**	**Age of TD youth, mean (range)**	**Quantity of CU by TD youth**	**Sample**	**Measures of CU or CUD traits**	**Time between MR scan and last CU**	**Comorbidity (% of CU youth with each condition)**	**Scanner strength (T)**	**FWHM (mm)**	***P*** **-value**	**Results**
Cousijn et al. (40)	Weekly CU^A^	33 (64%)	21.3 (18.0-25.0)	>10 days per month; 1579.5 (1425.0) joints lifetime use; duration use: 2.5(1.9) years	42 (62%)	21.9 (18.0-25.0)	<50 joints lifetime use	Community	TLFB; CUDIT	24 h abstinent; average abstinence in CU sample: 1.8 (2.3) days	TU (70%)	3.0T	8	ROI mask: *p* < 0.005 Whole Brain: *p* < 0.001, FWE: *p* < 0.05	Group-level analysis Heavy cannabis using adolescents had larger L/R anterior cerebellum volumes compared to Controls, but did not differ from controls in volumes of other brain regions. Correlation analysis Among heavy CU adolescents, amygdala and hippocampal volumes correlated negatively with the amount of cannabis use or problem-severity scores.
Gilman et al. (39)	Weekly ND-CU^B^	20 (45%)	21.3 (18.0-25.0)	>one use per week; 3.8 days/week; 11.2 joints/week; duration use: 6.21(3.43) years	20 (45%)	20.7 (18.0-25.0)	<5 use episodes lifetime; 0 use episodes in past 12 months	Community	TLFB; SCID DSM-IV	Overnight abstinence (> 12 hours)	OTU (70%) DTU (5%)	3.0T	6.9	Bonferroni Correction: (*p* < 0.05/4 = 0.0125)	Group-level analysis For GMV: MJ users had increased nucleus accumbens volumes compared to HC that reached trend-level. For GM density: MJ users had increased GM density in the left nucleus accumbens extending into the subcallosal cortex, hypothalamus, amygdala, and SL-extended amygdala after controlling for age, sex, alcohol use, and cigarette smoking.
Jarvis et al. (42)	BP-CUD^C^	7 (29%)	15 (12.0-18.0)	NR; all with CUD diagnosis; duration use: NP	BP: 7 (43%)	16 (12.0-19.0)	0	Clinical: Inpatient Psychiatric Unit	SCID DSM-IV, ASI, Substance Abuse Course-Modified Life II	NP; > 72 hours abstinent (based upon inpatient setting)	BP (100%)	3.0T	12	*P* ≤ 0.001, minimum cluster size 200 voxels	Group-level analyses: BP w/ CUD patients had decreased GMV in left fusiform gyrus and increased GMV in the right caudate and precentral gyrus and increased GM density in the right middle occipital gyrus, right fusiform gyrus, and cerebellar vermis compared to BP w/o CUD patients.
Orr et al. (12)	Low-level CU^D^	46 (65%)	14.6 (14–16)	1-2 instances of CU, lifetime; duration use: <1 year	46 (48%)	14.5 (14.0-16.0)	0	Community	ESPAD	NP; 13% of CU reported use of cannabis in past 7 days; 22% of CU reported use in past 30 days.	None	3.0T	8	*P* < 0.001,600 voxel cluster	Group-level analysis Low-level early-adolescent cannabis users had larger volumes in a number of brain regions compared to non-using age-matched controls. Low levels of cannabis use in cohort one was associated with greater gray matter volume in the hippocampus, amygdala, and striatum, bilateral parietal regions, cerebellum, and left middle temporal gyrus. Correlation analysis In addition, the magnitude of differences in GMV were associated with CB1 receptor availability from a separate dataset.
Thayer et al. (41)	Weekly CU^E^	201 (74%)	16 (14.0-18.0)	>one use per week; 20.6 (9.5) use days in past 30 days; duration use: NP	238 (66%)	16.0 (14.0-18.0	0	Juvenile Justice Involved	TLFB	NP	Alcohol Use	3.0T	6.9	Whole Brain: *P* < 0.001, 1000 voxel cluster	Group-level analysis No group-level differences in GMV were observed between CU and TD youth.
Weiland et al. (11)	Daily CU	50 (82%)	16.7 (14.0-18.0)	Daily use of cannabis; duration use: NP	50 (72%)	16.8 (14.0-18.0)	0	Juvenile Justice Involved	TLFB, past 90 days	NP	Alcohol Use	3.0T	6.9	Clusterwise extent correction: *t* > 2.3, F > 3.0	Group-level analysis There was no significant difference in any brain region between cannabis users and controls.

A *In Cousijn et al. (40), CU participants were heavy CU young adults defined as using cannabis 10 or more days in the month prior to assessment (i.e. > two times per week) and using cannabis on > 240 days over the 2 years prior to assessment*.

B *In Gilman et al. (39) – CU participants were defined as weekly or more frequent CU who did not meet DSM-IV criteria for cannabis dependence*.

C *In Jarvis et al. (42), all participants were diagnosed with Bipolar Disorder (BP) with CU participants also meeting criteria for DSM-IV diagnosis of current cannabis use disorder (CUD) based upon psychiatric interview (SU module of SCID, DSM-IV)*.

D *In Orr et al. (12), CU participants were low-frequency users defined as using cannabis on 1 or 2 instances in their lifetime based upon the ESPAD*.

E *In Thayer et al. (41) cannabis use was characterized as days of use in past 30 days from the TLFB with days use examined as a predictor of VBM outcomes in a combined sample of CU and non-using youth and in a sample restricted to participants using cannabis weekly or more frequently over the past 30 days. ^F^In Weiland et al. (11), CU participants were adolescents who used cannabis on a daily basis over the past 90 days*.

*ND-CU, Nondependent cannabis users; CU, cannabis users; BP, CUD Bipolar Disorder and CUD comorbidity; SCID (DSM-IV), Substance Use Disorder module of Structural Clinical Interview for Diagnostic and Statistical Manual Mental Disorders, 4th edition; ASI, Addictions Severity Index; ESPAD, European School Survey Project on Alcohol and Drugs; CUDIT, Cannabis Use Disorder Identification Test; TLFB, Timeline Follow Back; TU, Tobacco Users; OTU, Occasional Tobacco User; DTU, Daily Tobacco Users; NP, Not provided*.

### Meta-Analysis: Regional GMV Differences in CU vs. TD Youth

The primary SDM meta-analysis (not investigating age or sex) identified no regions showing significant GMV differences between youth with CU compared to TD youth. This null finding remained when analyses were rerun after from a restricted sample excluding the Jarvis et al. study of BP-CUD youth.

### Meta-Regression Analysis: Age-Related GMV Effects

**Figure 2:**
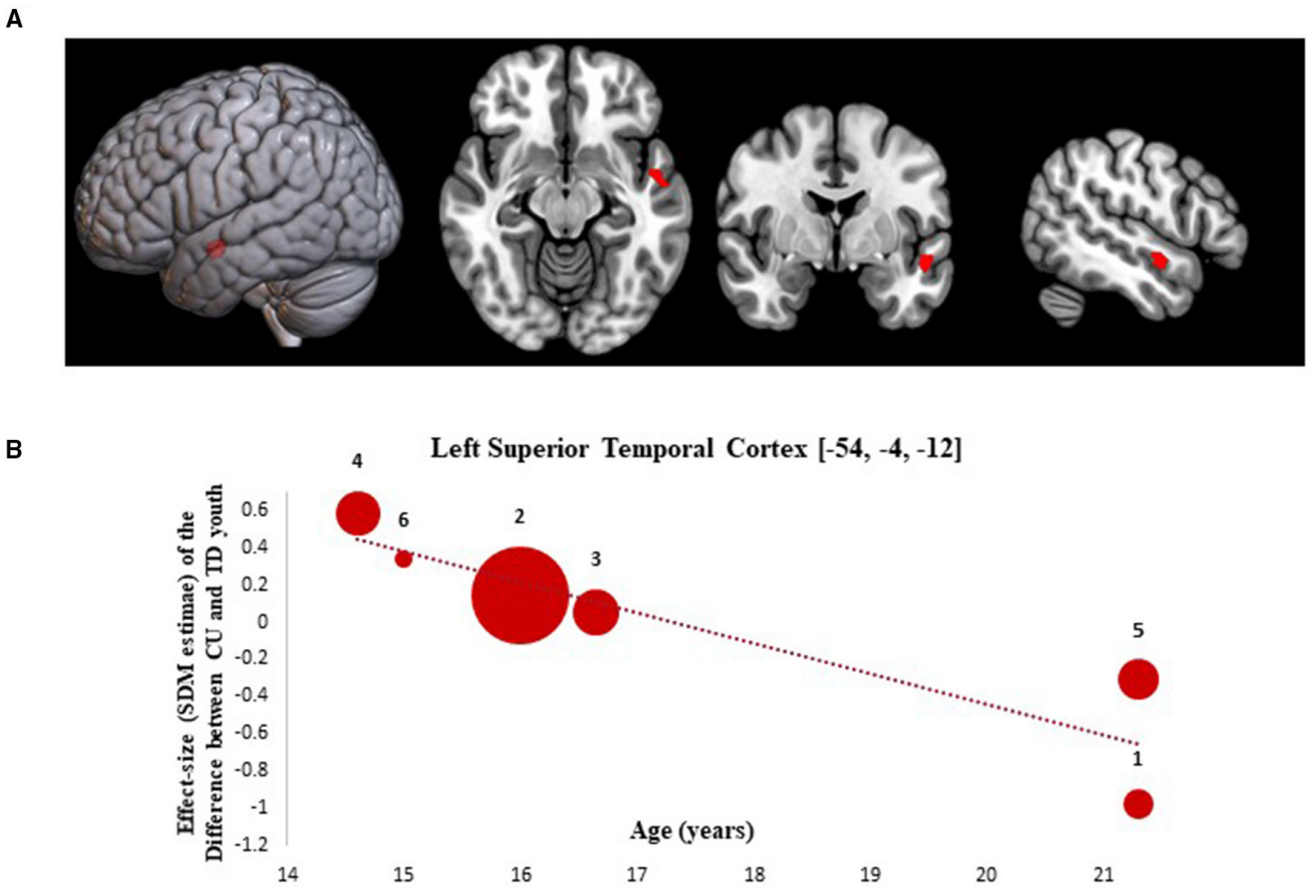
Meta-regression results showing associations between age at scan with gray matter differences between cannabis using and typically developing youth. Age-related meta-regression results. **(A)** Meta-regression results (CU > TD youth) showing associations between Age at Scan and gray matter differences between CU and TD youth shown in red. All results thresholded at *p* < 0.005. **(B)** Associations between age and gray matter differences in the left superior temporal cortex (85 voxels, SDM-Z = −3.168) (shown in red). Effect sizes (SDM-estimates) used to create the meta-regression plots were extracted from the peak of maximum slope significance. The meta-regression SDM-estimate value is derived from the proportion of studies that reported gray matter changes near the voxel so it is expected that some values are at 0 or near +/– 1. Each included study is represented as a numbered dot, with the dot size reflecting relative total sample size of each specific study in comparison to the average total sample size of all six studies included in the regression. Study key: 1 = Gilman et al. (39); 2 = Thayer et al. (41); 3 = Weiland et al. (11); 4 = Orr et al. (12); 5 = Cousijn et al. (40); 6 = Jarvis et al. (42).

Results from the SDM meta-regression examining the effect of age at time of scan on GMV differences between CU and TD youth are shown in Figure 2. The age-related meta-regression showed that increasing mean age across adolescence was associated with a relative decrease in GMV in youth with CU vs. age-matched TD youth in the left superior temporal gyrus (L-STG: 85 voxel cluster; MNI peak coordinate: x = −54, y = −4, z = −12; SDM Zmap = −3.168, *p* = 0.0008). This finding remained significant after repeating the main analysis following the removal of a single study in which both CU and TD participants had BP (42).

### Meta-Regression Analysis: Sex-Related GMV Effects

**Figure 3:**
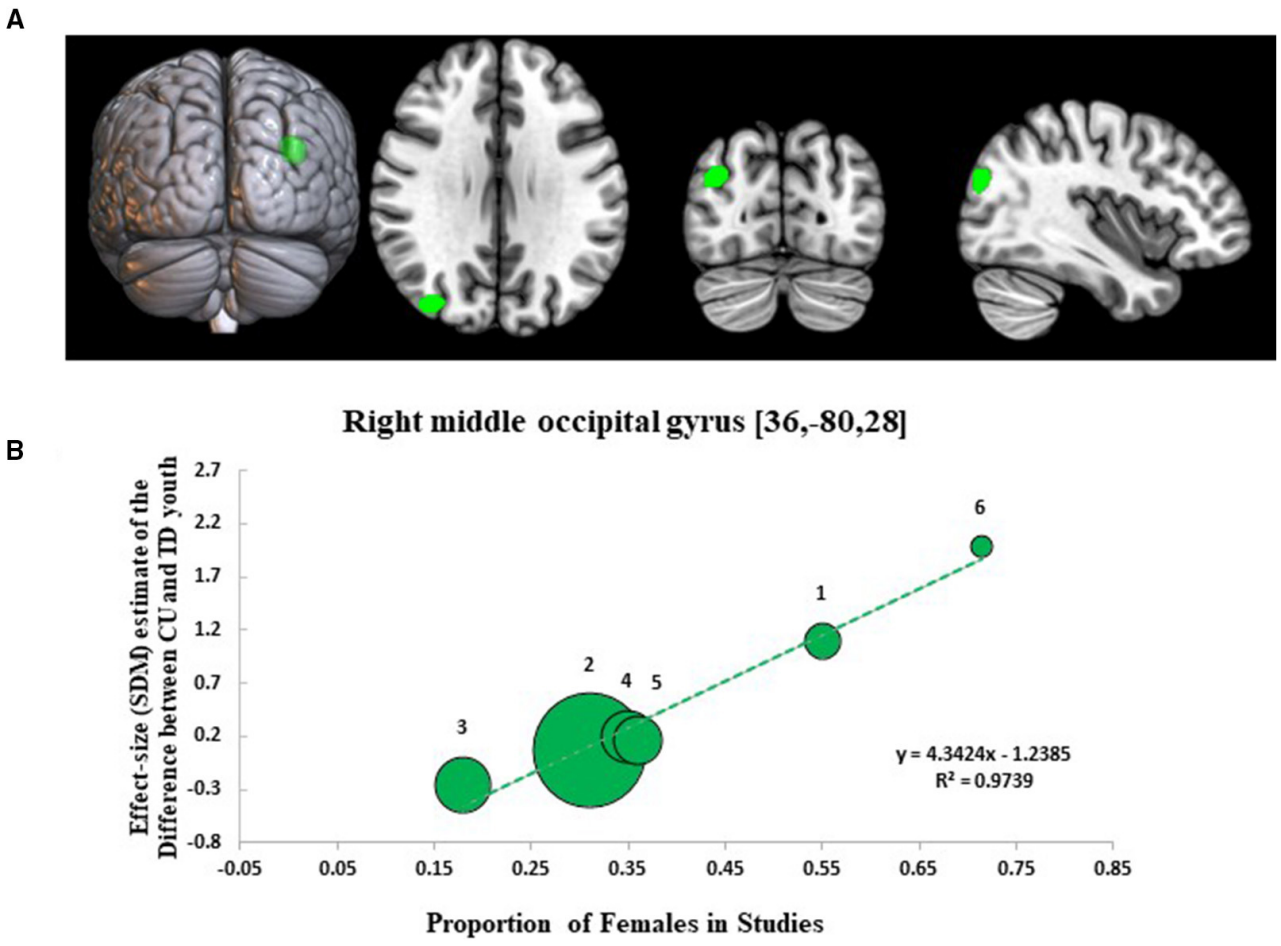
Meta-regression results showing associations between proportion of females in studies with gray matter differences between cannabis using and typically developing youth. Sex-related meta-regression results. **(A)** Meta-regression results (CU > TD youth) showing associations between proportion of females in studies and gray matter differences between CU and TD youth shown in green. All results thresholded at *p* < 0.005. **(B)** Associations between sex and gray matter differences in the right middle occipital gyrus (162 voxels, SDM-Z = 3.953) (shown in green). Effect sizes (SDM-estimates) used to create the meta-regression plots were extracted from the peak of maximum slope significance. The meta-regression SDM-estimate value is derived from the proportion of studies that reported gray matter changes near the voxel so it is expected that some values are at 0 or near +/– 1. Each included study is represented as a numbered dot, with the dot size reflecting relative total sample size of each specific study in comparison to the average total sample size of all six studies included in the regression. Study key: 1 = Gilman et al. (39); 2 = Thayer et al. (41); 3 = Weiland et al. (11); 4 = Orr et al. (12); 5 = Cousijn et al. (40); 6 = Jarvis et al. (42).

Results from the SDM meta-regression examining sex-dependent effects on GMV differences between CU and TD youth are shown in Figure 3. The sex-related meta-regression showed that increasing proportion of female participants in studies was associated with a relative increase in GMV in youth with CU compared to sex-matched TD youth in the right middle occipital gyrus (R-MOG: 162 voxel cluster; MNI peak coordinate: x = 36, y = −80, z = 28; SDM Zmap = 3.953, *p* = 0.00004). This finding was no longer significant following the removal of the Jarvis et al. study but remained significant after repeating the main analysis following the removal of each other study.

### Supplemental Analyses

As too few whole-brain VBM studies were identified for properly powered subgroup analyses, our planned a priori subgroup analyses were not conducted. Based upon the results from the main analysis which identified a significant age-related and sex-related GMV effects, we chose to still conduct our planned a priori supplemental meta-regression analyses examining the effect of other variables (recent CU frequency, duration of CU, and age range of studies) on GMV. These supplemental analyses are underpowered and should be interpreted as exploratory only. In supplemental meta-regression analyses, increasing duration of CU was associated with a relative decrease in GMV in the L-STG in CU vs. TD youth (Supplementary Figure S1: 145 voxel cluster; MNI peak coordinate: x = −52, y = −4, z = −14; SDM Zmap = −3.542, *p* = 0.0002). None of the other assessed variables were significantly associated with GMV differences between CU and TD youth in supplemental meta-regression analyses.

### Reliability Analysis

Jackknife sensitivity analysis of the primary meta-analytic results identified no additional significant clusters when studies were sequentially removed from the analysis. Jackknife sensitivity analyses of the meta-regression results (Supplementary Tables S4, S5) showed that age-related and sex-related GMV effects were largely preserved through most study combinations. Age-related GMV effects in the L-STG were preserved in four out of six study combinations and the sex-related GMV effects in R-MOG were preserved in five out of six study combinations. The L-STG cluster identified in the supplemental analyses showing GMV differences as a function of duration of CU was observed in four of the six studies (Supplementary Table S6).

## Discussion

The present meta-analysis investigated age-related and sex-related GMV differences between CU and TD youth to determine the influence of age and sex on reported cannabis-brain morphology relationships across adolescence. To our knowledge this is the first imaging-based meta-analysis of VBM studies of GMV to examine differences between CU and TD youth to specifically investigate for age-related and sex-related effects. The main findings were that CU youth (compared to TD youth) showed GMV differences in temporal and occipital regions that varied as a function of age and sex, respectively. When GMV differences were investigated without examining age or sex effects, no differences were observed between CU and TD youth. Across the six VBM studies included in the meta-analysis, there was significant heterogeneity noted in sample characteristics, comorbidity, and how CU was measured. Implications of these findings are discussed below.

Partially consistent with our hypotheses, we found evidence for age-related GMV differences between CU and TD youth in the L-STG but did not observe differences in other brain regions. This finding suggests that an age/developmental gradient effect of cannabis exposure across adolescence may exist. If true, an age gradient effect could explain some of the divergent results observed across studies. That age-related GMV differences in temporal regions are present in CU youth and decrease as a function of age is consistent with preclinical studies showing non-linear morphologic changes in CB1 receptor enriched brain regions following adolescent cannabis exposure (9, 10). Our results parallel prior human imaging studies showing increased volume and thickness in temporal regions of early-adolescent cannabis users and decreased volumes and thickness in temporal regions of late-adolescent and young adult cannabis users (12, 43, 44). In supplemental analyses, we also identified GMV differences in a L-STG cluster that varied as a function of duration of CU and showed significant overlap with the L-STG cluster identified in our age-related meta-regression analysis. Age and duration of use may be conflated in our analyses, especially as increasing age is associated with increased duration of CU among CU youth. As such, future studies with longitudinal prospective designs, such as the Adolescent Brain Cognitive Development (ABCD) study, are needed to disentangle the relative impact of changes in age and cannabis exposure effects on brain morphology. Our GMV results are consistent with a previous study showing that CUD status influences cortical maturation of the L-STG in adolescents with and without early-onset psychosis (EOP) who were initially scanned at age 16 and then again 18-months later (45). Cannabis exposure starting early and persisting throughout the middle-to-late adolescent periods is associated with greater cortical thinning in PFC regions by young adulthood (46). Moreover, greater duration of CU and higher cumulative cannabis exposure is associated with smaller volumes and thinner cortices in temporal and frontal regions of chronic CU adults who started using in early adolescence (15, 19, 20, 27).

Our findings should be considered within a developmental framework. Adolescence is a critical age range during which extensive cortical thinning and GM reductions occur (47). These morphologic changes are believed to represent normal maturational processes related to synaptic pruning (48). Given this, the age gradient effect hinted at by our results suggests the possibility that divergent structural abnormalities may result from cannabis exposure at different ages (e.g., early adolescence vs. young adulthood), and that cumulative cannabinoid exposure may also play a role in cannabis-brain morphology relationships. Further, this gradient could emerge as result of two distinct cannabis-related neuroadaptive/neurotoxic processes that shape cortical morphology in opposing ways at different times during development. For example, in early adolescence, a relative increase in L-STG volume in CU compared TD youth could reflect a disruption in synaptic pruning resulting in the preservation of synapses that would normally be eliminated during refinement of neural circuits (49). In contrast, during late adolescence/young adulthood a relative decrease in L-STG volume in CU compared to TD youth could reflect increased apoptotic mechanisms in specific neuronal cell bodies as a result of cannabis-induced neurotoxicity that occurs when cumulative cannabis exposure has exceeded a certain threshold (9). These developmental hypotheses require additional testing.

Of note, GMV effects related to age and duration of CU from our meta-regressions were both specific to the L-STG, a temporal region involved in auditory, speech, language, face, and emotion processing (50, 51). Temporal brain regions (such as the STG) have increased CB1 receptor expression compared to other cortical regions and thus may be more sensitive to cannabis exposure (33). Our findings are consistent with prior behavioral and functional MRI studies showing evidence of impairments in sensory gating and emotional face processing tasks and altered fMRI blood-oxygen-level-dependent (BOLD) response in the L-STG in CU youth (52–55). The finding also shows relevant overlap with sMRI studies in EOP and schizophrenia (SZD), where reduced gray matter in the L-STG has been observed among individuals with EOP and SZD compared to controls and is associated with increased severity of hallucinations and delusions (56, 57). This may carry clinical significance, especially given the growing literature showing that adolescent CU, especially with high Δ-9-THC potency chemotypes, is associated with increased risk for developing psychotic and affective disorders (58). As such, structural abnormalities in L-STG related to cannabis exposure could lead to impairments in social-cognitive processing, which, in turn could increase the risk for psychotic and affective symptoms in CU youth. Based upon our results, additional research is warranted to investigate the potential role that L-STG abnormalities play in psychosis and negative emotionality of CU youth, as this work may improve our understanding of cannabis's contribution to neurodevelopmental risk factors for psychotic and affective disorders in young people.

One of the main objectives of this study was to investigate the influence of sex distribution on GMV differences between CU and TD youth to determine if any GMV effects are sex-dependent. We identified an occipital cluster centered in the R-MOG that varied between CU and TD youth as a function of sex distribution showing increased GMV in CU compared to TD youth in studies that had a higher proportion of female participants and the opposite relationship (decreased GMV) in studies that had a higher proportion of male participants. This suggests that sex may moderate the relationship between cannabis exposure and occipital morphology during adolescence. Our results regarding sex-related GMV effects are consistent with prior studies in CU adolescents and adults that have shown differences in GMV, cortical thickness, and gyrification in women that are directionally opposition from those found in men (26, 34, 35, 59, 60). A number of possible factors could explain this result. Sex differences in the effect of adolescent cannabis exposure on occipital morphology could result from sexual dimorphism of endocannabinoid system (eCB) tonic signaling (61), CNS signaling pathways (62), hormonal influences (63), or pharmacokinetics (64). Additionally, they could reflect sex differences in brain age at time of cannabis exposure, given that adolescent girls brains are at a more advanced stage of maturation compared to age-matched boys (47). Based upon this, increased R-MOG volume in CU girls relative to sex-matched controls could be related to disruptions in synaptic pruning (49) and decreased R-MOG volume in CU boys could reflect increased sensitivity to cannabis-related neurotoxicity via apoptotic-mechanisms (9). Alternatively, this finding could reflect general neurodevelopmental differences between boys and girls, although this is less likely as sex differences in adolescent brain morphology are less pronounced in occipital regions (65, 66). The sex-related GMV effect could also be the result of differences in cannabis-related behavioral phenotypes between boys and girls who use cannabis. Adolescent boys initiate cannabis earlier than girls, and adolescent girls who use cannabis may have more cannabis-related problems and higher rates of co-occurring/comorbid affective symptoms and disorders, with all of these factors potentially impacting brain morphology (28).

The MOG is involved in visual information processing, attention, and affective and cognitive bias processing (67), which may be dysfunctional in CU individuals (68). Thus, our findings showing sex-related structural abnormalities in the MOG might underlie impairment in these neurocognitive processes and relate to the expression of increased cannabis-related problems and comorbid affective disorders in CU adolescent girls. This interpretation is supported by evidence from previous fMRI studies showing altered BOLD fMRI response in the R-MOG of CU adolescents and adults during visuospatial memory and attentional tasks (69–71) and a previous sMRI study showing cannabis-related changes cortical thickness in the occipital lobe of patients with EOP (56). The result also fits well with fMRI studies reporting alterations in BOLD fMRI response in the MOG and functional connectivity (FC) between the MOG and the thalamus, PFC, and hippocampus in adults with obsessive compulsive disorder (72) and women with depression (73). Moreover, the latter of these two findings points to possible sex differences in relation to MOG activity and connectivity in depressed women. This line of research warrants further study.

Regarding our main findings, it is important to note that our age- and sex-related GMV results showed modest effect sizes and were not replicated in the primary GMV meta-analysis. Given this, it is important to interpret these results cautiously. The age- and sex-related GMV findings could reflect true but subtle differences between CU and TD youth, or alternately could index individual differences in morphology that approximate the range of normal variability which is higher during development (11). Subtle morphological differences related to cannabis exposure, if present, could be obfuscated in studies that are underpowered or have a broad age-range or skewed sex distribution. Problematically, studies in the extant literature without these limitations are rare. Multiple genetic and environmental factors may contribute variance to neuroanatomical abnormalities observed in CU youth. Age- and sex-related GMV differences between CU and TD youth could predate cannabis exposure and be attributed to common predispositional factors, or alternatively could emerge following exposure as a result of cannabis-induced neuroadaptive changes. These explanations are not mutually exclusive. In fact, recent evidence has emerged that partially supports both models [e.g., shared genetic factors (74); premorbid OPFC volumes predicting cannabis initiation in adolescence (75); and cannabis-induced neuroadaptive changes (44, 46)] suggesting complex bidirectional relationships. The ongoing ABCD study should aid in clarifying the nature, directionality, and mediators and moderators of cannabis-brain morphology relationships emerging during adolescence. In addition to the ABCD study, other imaging-treatment studies should also be conducted to address more focal questions about the predictive capacity of neurobehavioral variables on CUD treatment outcomes and the moderating role of sex, age, and other clinical variables (comorbidity, polydrug use) as these types of studies may inform the development of sex-specific treatments and treatment matching algorithms in the future.

This meta-analytic report has a number of important limitations. As the study was a meta-analysis, it was reliant on the study methodology, analytic approaches, and assessments done in each of the VBM studies, few of which were designed or powered to answer specific research questions about age- and sex-related differences in brain morphometry. Based upon study heterogeneity, lack of sufficient information on experimental design and analyses reported by some studies, and the large number of studies using ROI-based analyses, we were limited to making inferences from published coordinates and the number of eligible studies for inclusion in the primary meta-analysis and meta-regressions was small (*n* = 6 studies). As such, our main analyses may have been under powered to detect subtle neuroanatomical differences with small effect sizes and there were insufficient number of studies to conduct appropriately powered subgroup analyses. We sought to address these issues by contacting authors and examining repositories for unthresholded statistical maps with the goal of expanding the number of included studies, but were unsuccessful. Changes in data management and reporting practices, including expectations for sharing of unthresholded statistical maps or full datasets in online repositories, are needed to support meta-analytic inquiry in this still emerging field. The limited number of studies identified for our meta-analysis also limited our ability to conduct planned sensitivity analyses controlling for alcohol and tobacco co-use. This is problematic, as many CU youth co-use alcohol and tobacco products and recent studies suggest that co-use of cannabis, alcohol, and tobacco may interact and produce unique neuroanatomic and functional abnormalities in poly-users compared to mono-users of these drugs (76, 77). As such, future neuroimaging studies should seek to include poly- and mono-users to dissociate distinct and overlapping effects cannabis, alcohol, and tobacco on brain development in youth. Given the focus on VBM studies, our results are inherently linked to the limitations of this sMRI analytic technique, including its weakness in detecting spatially complex group-level differences such as gyrification and microstructure. Still, it should be noted that our findings overlap with the results from sMRI studies in CU youth and adults measuring cortical thickness, surface area, and microstructural variation (39, 44, 78). Recent studies suggest divergent effects of youth CU on brain and health outcomes as a function of age of cannabis initiation (5, 20). As such, our decision to set the age window broadly (12-21 years) and to include studies with young adult samples could also be viewed as a limitation, although a necessary one, given the small number of whole-brain GMV studies identified for inclusion in the meta-analysis. As observable from Figure 2, including youth through age 21 years added variance to the GMV results. This may have obfuscated a main effect of cannabis exposure on GMV, if one was present, but also enabled the examination of GMV effects related to CU as a function of age, sex, and other demographic and clinical variables (which were heterogenous across samples), resulting in the identification of novel age-related and sex-related GMV effects in CU vs. TD youth. Future population-based longitudinal studies should investigate cannabis exposure effects between subjects across narrow age bands (12-14 years, 15-17 years, 18-19 years, 20-21 years) and within subjects over time to identify critical periods of vulnerability to cannabis exposure and to characterize the impact of cannabis exposure across adolescence on brain growth trajectories. Another major limitation is the lack of biochemical quantification of cannabis exposure, and specifically of Δ-9-THC and cannabidiol (CBD) levels, in studies included in this meta-analysis. This limited our ability to investigate this relevant domain. Given preliminary data showing divergent and at times opposing effects of Δ-9-THC and CBD on brain structure and function in adults (79), future studies should measure Δ-9-THC and CBD exposure from cannabis product use and relate these exposures to brain changes in CU youth. Lastly, the majority of studies used in the present meta-analytic report used cross-sectional designs precluding the ability to assign causal determinations. As the field grows and more studies are published using standardized neuroimaging methods and longitudinal designs, quantitative meta-analyses of these studies looking for convergent findings will further inform our understanding of the neurobiological effects of adolescent cannabis exposure. Despite these limitations, the study also has notable strengths. It is one of the first meta-analytic studies to examine neurobiological correlates of adolescent CU. As such, it identifies key targets to guide future research and theory development. Additional strengths include its use of SDM meta-analytic/meta-regression techniques and focus on quantitative assessment of the relationships between age, sex, cannabis exposure, and brain morphology in a developmental sample.

## Conclusions

In conclusion, the results of this meta-analysis suggest that CU youth have significantly reduced GMV in the L-STG and increased GMV in the R-MOG that vary as a function of age and sex, respectively. Duration of cannabis exposure was also associated with reduced L-STG GMV. These findings help to build a more coherent picture of structural alterations in CU youth and how factors such as age and sex influence the presentation of GMV alterations in this population. Our results lend further support to the hypothesis that adolescent cannabis exposure alters brain growth trajectories in subtle ways, and highlights the need for large-scale prospective longitudinal studies to further probe cannabis-brain morphology relationships.

## Data Availability Statement

The raw data supporting the conclusions of this article will be made available by the authors, without undue reservation.

## Author Contributions

AA: conceptualization, methodology, investigation, formal analysis, data curation, visualization, writing—original draft, and review and editing. GP: methodology, systematic review, and writing—review and editing. KK: data curation, visualization, and writing—review and editing. MV: visualization and writing—review and editing. KR: methodology, systematic review, data curation, visualization, and writing—review and editing. RL and JN: systematic review methodology, investigation, data collection and curation, and writing—review and editing. CH: conceptualization, methodology, investigation, formal analysis, data curation, writing—original draft, review and editing, and supervision. All authors contributed to the article and approved the submitted version.

## Funding

This work was supported by an AACAP Career Development Award for Adolescent Substance Abuse (K12DA000357) and from a Doris Duke Charitable Foundation Early Career Investigator Award.

## Conflict of Interest

CH receives grant support from the NIH (K12DA000357), SAMHSA (H79 SP082126), Doris Duke Charitable Foundation, AACAP, National Network of Depression Centers, and Johns Hopkins University, and serves as a SAMHSA subject matter expert related to co-occurring substance use disorders and severe emotional disturbance in youth. The remaining authors declare that the research was conducted in the absence of any commercial or financial relationships that could be construed as a potential conflict of interest.

## Publisher's Note

All claims expressed in this article are solely those of the authors and do not necessarily represent those of their affiliated organizations, or those of the publisher, the editors and the reviewers. Any product that may be evaluated in this article, or claim that may be made by its manufacturer, is not guaranteed or endorsed by the publisher.

## References

[1] SAMHSA. Key Substance Use and Mental Health Indicators in the United States: results from the 2016 National Survey on Drug Use and Health (HHS Publication No. SMA 17-5044, NSDUH Series H-52). Rockville, MD: Substance Abuse Mental Health Services Administration, Center for Behavioral Health Statistics and Quality (2018).

[2] SAMHSA. Treatment Episode Data Set (TEDS): 2017. Admissions to and Discharges from Publicly-Funded Substance Use Treatment. Rockville, MD: Substance Abuse Mental Health Services Administration, Center for Behavioral Health Statistics and Quality (2019).

[3] Gentzke AS Creamer M Cullen KA Ambrose BK Willis G Jamal A . Vital signs: tobacco product use among middle and high school students - United States, 2011-2018. MMWR Morb Mortal Wkly Rep. (2019) 68:157–64. 10.15585/mmwr.mm6806e1 30763302 PMC6375658

[4] Johnston Lloyd D Miech Richard A O'Malley Patrick M Bachman Jerald G Schulenberg John E Patrick Megan E. Monitoring the Future National Survey Results on Drug Use, 1975-2017: Overview, Key Findings on Adolescent Drug Use. Ann Arbor: Institute of Social Research Michigan (2018). 10.3998/2027.42/148123

[5] Hammond CJ Chaney A Hendrickson B Sharma P. Cannabis use among US. Adolescents in the era of marijuana legalization: a review of changing use patterns, comorbidity, and health correlates. Int Rev Psychiatry. (2020) 32:221–34. 10.1080/09540261.2020.1713056 32026735 PMC7588219

[6] Moore TH Zammit S Lingford-Hughes A Barnes TR Jones PB Burke M . Cannabis use and risk of psychotic or affective mental health outcomes: a systematic review. Lancet. (2007) 370:319–28. 10.1016/S0140-6736(07)61162-3 17662880

[7] Silins E Horwood LJ Patton GC Fergusson DM Olsson CA Hutchinson DM . Young adult sequelae of adolescent cannabis use: an integrative analysis. Lancet Psychiatry. (2014) 1:286–93. 10.1016/S2215-0366(14)70307-4 26360862

[8] Hammond CJ Mayes LC Potenza MN. Neurobiology of adolescent substance use and addictive behaviors: treatment implications. Adolesc Med State Art Rev. (2014) 25:15–32. 25022184 PMC4446977

[9] Chye Y Kirkham R Lorenzetti V McTavish E Solowij N Yücel M. Cannabis, cannabinoids, and brain morphology: a review of the evidence. Biol Psychiatry Cogn Neurosci Neuroimaging. (2021) 6:627–35. 10.1016/j.bpsc.2020.07.009 32948510

[10] Panlilio LV Justinova Z. Preclinical studies of cannabinoid reward, treatments for cannabis use disorder, and addiction-related effects of cannabinoid exposure. Neuropsychopharmacology. (2018) 43:116–41. 10.1038/npp.2017.193 28845848 PMC5719102

[11] Weiland BJ Thayer RE Depue BE Sabbineni A Bryan AD Hutchison KE. Daily marijuana use is not associated with brain morphometric measures in adolescents or adults. J Neurosci. (2015) 35:1505–12. 10.1523/JNEUROSCI.2946-14.2015 25632127 PMC4308597

[12] Orr C Spechler P Cao Z Albaugh M Chaarani B Mackey S . Grey matter volume differences associated with extremely low levels of cannabis use in adolescence. J Neurosci. (2019) 39:1817–27. 10.1523/JNEUROSCI.3375-17.2018 30643026 PMC6407302

[13] Maple KE Thomas AM Kangiser MM Lisdahl KM. Anterior cingulate volume reductions in abstinent adolescent and young adult cannabis users: association with affective processing deficits. Psychiatry Res Neuroimaging. (2019) 288:51–9. 10.1016/j.pscychresns.2019.04.011 31079000 PMC6548454

[14] Sagar KA Gruber SA. Marijuana matters: reviewing the impact of marijuana on cognition, brain structure and function, and exploring policy implications and barriers to research. Int Rev Psychiatry. (2018) 30:251–67. 10.1080/09540261.2018.1460334 29966459 PMC6455965

[15] Battistella G Fornari E Annoni JM Chtioui H Dao K Fabritius M . Long-term effects of cannabis on brain structure. Neuropsychopharmacology. (2014) 39:2041–8. 10.1038/npp.2014.67 24633558 PMC4104335

[16] Chye Y Suo C Lorenzetti V Batalla A Cousijn J Goudriaan AE . Cortical surface morphology in long-term cannabis users: a multi-site MRI study. Eur Neuropsychopharmacol. (2019) 29:257–65. 10.1016/j.euroneuro.2018.11.1110 30558823

[17] Chye Y Suo C Yücel M den Ouden L Solowij N Lorenzetti V. Cannabis-related hippocampal volumetric abnormalities specific to subregions in dependent users. Psychopharmacology. (2017) 234:2149–57. 10.1007/s00213-017-4620-y 28424833

[18] Filbey FM Dunlop J. Differential reward network functional connectivity in cannabis dependent and non-dependent users. Drug Alcohol Depend. (2014) 140:101–11. 10.1016/j.drugalcdep.2014.04.002 24838032 PMC4349558

[19] Filbey FM Aslan S Calhoun VD Spence JS Damaraju E Caprihan A . Long-term effects of marijuana use on the brain. Proc Natl Acad Sci USA. (2014) 111:16913–8. 10.1073/pnas.1415297111 25385625 PMC4250161

[20] Filbey FM McQueeny T DeWitt SJ Mishra V. Preliminary findings demonstrating latent effects of early adolescent marijuana use onset on cortical architecture. Dev Cogn Neurosci. (2015) 16:16–22. 10.1016/j.dcn.2015.10.001 26507433 PMC4691364

[21] Lorenzetti V Chye Y Silva P Solowij N Roberts CA. Does regular cannabis use affect neuroanatomy? An updated systematic review and meta-analysis of structural neuroimaging studies. Eur Arch Psychiatry Clin Neurosci. (2019) 269:59–71. 10.1007/s00406-019-00979-1 30706169

[22] Tzilos GK Cintron CB Wood JBR Simpson NS Young AD Pope HG . Lack of hippocampal volume change in long-term heavy cannabis users. Am J Addict. (2005) 14:64–72. 10.1080/10550490590899862 15804878

[23] Pando-Naude V Toxto S Fernandez-Lozano S Parsons CE Alcauter S Garza-Villarreal EA . Gray and white matter morphology in substance use disorders: a neuroimaging systematic review and meta-analysis. Transl Psychiatry. (2021) 11:29. 10.1038/s41398-020-01128-2 33431833 PMC7801701

[24] Chye Y Christensen E Yücel M. Cannabis use in adolescence: a review of neuroimaging findings. J Dual Diagn. (2020) 16:83–105. 10.1080/15504263.2019.1636171 31311489

[25] Jacobus J Squeglia LM Sorg SF Nguyen-Louie TT Tapert SF. Cortical thickness and neurocognition in adolescent marijuana and alcohol users following 28 days of monitored abstinence. J Stud Alcohol Drugs. (2014) 75:729–43. 10.15288/jsad.2014.75.729 25208190 PMC4161693

[26] Medina KL McQueeny T Nagel BJ Hanson KL Yang TT Tapert SF. Prefrontal cortex morphometry in abstinent adolescent marijuana users: subtle gender effects. Addict Biol. (2009) 14:457–68. 10.1111/j.1369-1600.2009.00166.x 19650817 PMC2741544

[27] Ashtari M Avants B Cyckowski L Cervellione KL Roofeh D Cook P . Medial temporal structures and memory functions in adolescents with heavy cannabis use. J Psychiatr Res. (2011) 45:1055–66. 10.1016/j.jpsychires.2011.01.004 21296361 PMC3303223

[28] Calakos KC Bhatt S Foster DW Cosgrove KP. Mechanisms underlying sex differences in cannabis use. Curr Addict Rep. (2017) 4:439–53. 10.1007/s40429-017-0174-7 29503790 PMC5828236

[29] Hernandez-Avila CA Rounsaville BJ Kranzler HR. Opioid-, cannabis- and alcohol-dependent women show more rapid progression to substance abuse treatment. Drug Alcohol Depend. (2004) 74:265–72. 10.1016/j.drugalcdep.2004.02.001 15194204

[30] Herrmann ES Weerts EM Vandrey R. Sex differences in cannabis withdrawal symptoms among treatment-seeking cannabis users. Exp Clin Psychopharmacol. (2015) 23:415–21. 10.1037/pha0000053 26461168 PMC4747417

[31] Sherman BJ McRae-Clark AL Baker NL Sonne SC Killeen TK Cloud K . Gender differences among treatment-seeking adults with cannabis use disorder: Clinical profiles of women and men enrolled in the achieving cannabis cessation-evaluating N-acetylcysteine treatment (ACCENT) study. Am J Addict. (2017) 26:136–44. 10.1111/ajad.12503 28152236 PMC5323358

[32] Fattore L Spano MS Altea S Fadda P Fratta W. Drug- and cue-induced reinstatement of cannabinoid-seeking behaviour in male and female rats: influence of ovarian hormones. Br J Pharmacol. (2010) 160:724–35. 10.1111/j.1476-5381.2010.00734.x 20590575 PMC2931571

[33] Farquhar CE Breivogel CS Gamage TF Gay EA Thomas BF Craft RM . Sex, THC, and hormones: effects on density and sensitivity of CB(1) cannabinoid receptors in rats. Drug Alcohol Depend. (2019) 194:20–7. 10.1016/j.drugalcdep.2018.09.018 30391834 PMC6312486

[34] McQueeny T Padula CB Price J Medina KL Logan P Tapert SF. Gender effects on amygdala morphometry in adolescent marijuana users. Behav Brain Res. (2011) 224:128–34. 10.1016/j.bbr.2011.05.031 21664935 PMC3139567

[35] Rossetti MG Mackey S Patalay P Allen NB Batalla A Bellani M . Sex and dependence related neuroanatomical differences in regular cannabis users: findings from the ENIGMA Addiction Working Group. Transl Psychiatry. (2021) 11:272. 10.1038/s41398-021-01382-y 33958576 PMC8102553

[36] Price JS McQueeny T Shollenbarger S Browning EL Wieser J Lisdahl Krista M. Effects of marijuana use on prefrontal and parietal volumes and cognition in emerging adults. Psychopharmacology. (2015) 232:2939–50. 10.1007/s00213-015-3931-0 25921032 PMC4533900

[37] Radua J Mataix-Cols D Phillips ML El-Hage W Kronhaus DM Cardoner N . A new meta-analytic method for neuroimaging studies that combines reported peak coordinates and statistical parametric maps. Eur Psychiatry. (2012) 27:605–11. 10.1016/j.eurpsy.2011.04.001 21658917

[38] Moher D Liberati A Tetzlaff J Altman DG. Preferred reporting items for systematic reviews and meta-analyses: the PRISMA statement. BMJ. (2009) 339:b2535. 10.1136/bmj.b2535 19622551 PMC2714657

[39] Gilman JM Kuster JK Lee S Lee MJ Kim BW Makris N . Cannabis use is quantitatively associated with nucleus accumbens and amygdala abnormalities in young adult recreational users. J Neurosci. (2014) 34:5529–38. 10.1523/JNEUROSCI.4745-13.2014 24741043 PMC3988409

[40] Cousijn J Wiers RW Ridderinkhof KR van den Brink W Veltman DJ Goudriaan AE. Grey matter alterations associated with cannabis use: results of a VBM study in heavy cannabis users and healthy controls. Neuroimage. (2012) 59:3845–51. 10.1016/j.neuroimage.2011.09.046 21982932

[41] Thayer RE York Williams S Karoly HC Sabbineni A Ewing SF Bryan AD Hutchison KE. Structural neuroimaging correlates of alcohol and cannabis use in adolescents and adults. Addiction. (2017) 112:2144–54. 10.1111/add.13923 28646566 PMC5673530

[42] Jarvis K DelBello MP Mills N Elman I Strakowski SM Adler CM. Neuroanatomic comparison of bipolar adolescents with and without cannabis use disorders. J Child Adolesc Psychopharmacol. (2008) 18:557–63. 10.1089/cap.2008.033 19108660 PMC2692725

[43] Gruber SA Dahlgren Mary K Sagar KA Gönenc A Killgore WDS. Age of onset of marijuana use impacts inhibitory processing. Neurosci Lett. (2012) 511:89–94. 10.1016/j.neulet.2012.01.039 22306089 PMC3659423

[44] Jacobus J Squeglia LM Meruelo AD Castro N Brumback T Giedd JN . Cortical thickness in adolescent marijuana and alcohol users: a three-year prospective study from adolescence to young adulthood. Dev Cogn Neurosci. (2015) 16:101–9. 10.1016/j.dcn.2015.04.006 25953106 PMC4624050

[45] Epstein KA Kumra S. Altered cortical maturation in adolescent cannabis users with and without schizophrenia. Schizophr Res. (2015) 162:143–52. 10.1016/j.schres.2014.11.029 25600549

[46] Albaugh MD Ottino-Gonzalez J Sidwell A Lepage C Juliano A Owens MM . Association of cannabis use during adolescence with neurodevelopment. JAMA Psychiatry. (2021) 78:1–11. 10.1001/jamapsychiatry.2021.1258 34132750 PMC8209561

[47] Gogtay N Giedd JN Lusk L Hayashi KM Greenstein D Vaituzis AC . Dynamic mapping of human cortical development during childhood through early adulthood. Proc Natl Acad Sci USA. (2004) 101:8174–9. 10.1073/pnas.0402680101 15148381 PMC419576

[48] Sakai J. Core concept: how synaptic pruning shapes neural wiring during development and, possibly, in disease. Proc Natl Acad Sci USA. (2020) 117:16096–9. 10.1073/pnas.2010281117 32581125 PMC7368197

[49] Hoffman AF Hwang EK Lupica CR. Impairment of synaptic plasticity by cannabis, Δ(9)-THC, synthetic cannabinoids. Cold Spring Harb Perspect Med. (2021) 11:a039743. 10.1101/cshperspect.a039743 32341064 PMC8091957

[50] Bigler ED Mortensen S Neeley ES Ozonoff S Krasny L Johnson M . Superior temporal gyrus, language function, and autism. Dev Neuropsychol. (2007) 31:217–38. 10.1080/87565640701190841 17488217

[51] Celsis P Boulanouar K Doyon B Ranjeva JP Berry I Nespoulous JL . Differential fMRI responses in the left posterior superior temporal gyrus and left supramarginal gyrus to habituation and change detection in syllables and tones. Neuroimage. (1999) 9:135–44. 10.1006/nimg.1998.0389 9918735

[52] Aloi J Blair KS Crum KI Meffert H White SF Tyler PM . Adolescents show differential dysfunctions related to alcohol and cannabis use disorder severity in emotion and executive attention neuro-circuitries. Neuroimage Clin. (2018) 19:782–92. 10.1016/j.nicl.2018.06.005 29988822 PMC6031867

[53] Blest-Hopley G Giampietro V Bhattacharyya S. Residual effects of cannabis use in adolescent and adult brains - a meta-analysis of fMRI studies. Neurosci Biobehav Rev. (2018) 88:26–41. 10.1016/j.neubiorev.2018.03.008 29535069

[54] Nestor L Roberts G Garavan H Hester R. Deficits in learning and memory: parahippocampal hyperactivity and frontocortical hypoactivity in cannabis users. NeuroImage. (2008) 40:1328–39. 10.1016/j.neuroimage.2007.12.059 18296071

[55] Skosnik PD Cortes-Briones JA Hajos M. It's all in the rhythm: the role of cannabinoids in neural oscillations and psychosis. Biol Psychiatry. (2016) 79:568–77. 10.1016/j.biopsych.2015.12.011 26850792

[56] Rais M van Haren NE Cahn W Schnack HG Lepage C Collins L . Cannabis use and progressive cortical thickness loss in areas rich in CB1 receptors during the first five years of schizophrenia. Eur Neuropsychopharmacol. (2010) 20:855–65. 10.1016/j.euroneuro.2010.08.008 20863671

[57] Takahashi T Suzuki M Zhou SY Tanino R Hagino H Kawasaki Y . Morphologic alterations of the parcellated superior temporal gyrus in schizophrenia spectrum. Schizophr Res. (2006) 83:131–43. 10.1016/j.schres.2006.01.016 16503399

[58] Di Forti MM Craig D Paola P Carmine M Valeria M Murray RM . High-potency cannabis and the risk of psychosis. Br J Psychiatry. (2009) 195:488–91. 10.1192/bjp.bp.109.064220 19949195 PMC2801827

[59] McPherson KL Tomasi DG Wang GJ Manza P Volkow ND. Cannabis affects cerebellar volume and sleep differently in men and women. Front Psychiatry. (2021) 12:643193. 10.3389/fpsyt.2021.643193 34054601 PMC8155508

[60] Sullivan RM Wallace AL Wade NE Swartz AM Lisdahl KM. Assessing the role of cannabis use on cortical surface structure in adolescents and young adults: exploring gender and aerobic fitness as potential moderators. Brain Sci. (2020) 10:117. 10.3390/brainsci10020117 32098300 PMC7071505

[61] Melis M De Felice M Lecca S Fattore L Pistis M. Sex-specific tonic 2-arachidonoylglycerol signaling at inhibitory inputs onto dopamine neurons of Lister Hooded rats. Front Integr Neurosci. (2013) 7:93. 10.3389/fnint.2013.00093 24416004 PMC3867690

[62] Rosas M Porru S Giugliano V Antinori S Scheggi S Fadda P . Sex-specific differences in cannabinoid-induced extracellular-signal-regulated kinase phosphorylation in the cingulate cortex, prefrontal cortex, and nucleus accumbens of Lister Hooded rats. Behav Pharmacol. (2018) 29:473–81. 10.1097/FBP.0000000000000395 29595540

[63] Cooper ZD Craft RM. Sex-dependent effects of cannabis and cannabinoids: a translational perspective. Neuropsychopharmacology. (2018) 43:34–51. 10.1038/npp.2017.140 28811670 PMC5719093

[64] Tseng AH Harding JW Craft RM. Pharmacokinetic factors in sex differences in Delta 9-tetrahydrocannabinol-induced behavioral effects in rats. Behav Brain Res. (2004) 154:77–83. 10.1016/j.bbr.2004.01.029 15302113

[65] Koolschijn P Cédric MP Crone EA. Sex differences and structural brain maturation from childhood to early adulthood. Dev Cogn Neurosci. (2013) 5:106–18. 10.1016/j.dcn.2013.02.003 23500670 PMC6987760

[66] Lenroot Rhoshel K Giedd Jay N. Sex differences in the adolescent brain. Brain Cogn. (2010) 72:46–55. 10.1016/j.bandc.2009.10.008 19913969 PMC2818549

[67] Vuilleumier P Driver J. Modulation of visual processing by attention and emotion: windows on causal interactions between human brain regions. Philo Trans Royal Soc London B Biol Sci. (2007) 362:837–55. 10.1098/rstb.2007.2092 17395574 PMC2430001

[68] Bloomfield MAP Hindocha C Sebastian FG Wall MB Lees R Petrilli K . The neuropsychopharmacology of cannabis: a review of human imaging studies. Pharmacol Ther. (2019) 195:132–61. 10.1016/j.pharmthera.2018.10.006 30347211 PMC6416743

[69] Lopez-Larson MP Rogowska J Bogorodzki P Bueler CE McGlade EC Yurgelun-Todd DA. Cortico-cerebellar abnormalities in adolescents with heavy marijuana use. Psychiatry Res. (2012) 202:224–32. 10.1016/j.pscychresns.2011.11.005 22835865 PMC3423594

[70] Schweinsburg AD Nagel BJ Schweinsburg BC Park A Theilmann RJ Tapert SF. Abstinent adolescent marijuana users show altered fMRI response during spatial working memory. Psychiatry Res. (2008) 163:40–51. 10.1016/j.pscychresns.2007.04.018 18356027 PMC2832586

[71] Tapert SF Schweinsburg AD Drummond SP Paulus MP Brown SA Yang TT . Functional MRI of inhibitory processing in abstinent adolescent marijuana users. Psychopharmacology. (2007) 194:173–83. 10.1007/s00213-007-0823-y 17558500 PMC2269705

[72] Li K Zhang H Yang Y Zhu J Wang B Shi Y . Abnormal functional network of the thalamic subregions in adult patients with obsessive-compulsive disorder. Behav Brain Res. (2019) 371:111982. 10.1016/j.bbr.2019.111982 31141727

[73] Teng C Zhou J Ma H Tan Y Wu X Guan C . Abnormal resting state activity of left middle occipital gyrus and its functional connectivity in female patients with major depressive disorder. BMC Psychiatry. (2018) 18:370. 10.1186/s12888-018-1955-9 30477561 PMC6258168

[74] Pagliaccio D Barch DM Bogdan R Wood PK Lynskey MT Heath AC . Shared predisposition in the association between cannabis use and subcortical brain structure. JAMA Psychiatry. (2015) 72:994–1001. 10.1001/jamapsychiatry.2015.1054 26308883 PMC4624286

[75] Cheetham A Allen NB Whittle S Simmons JG Yücel M Lubman DI. Orbitofrontal volumes in early adolescence predict initiation of cannabis use: a 4-year longitudinal and prospective study. Biol Psychiatry. (2012) 71:684–92. 10.1016/j.biopsych.2011.10.029 22129756

[76] Hammond CJ Wu J Krishnan-Sarin S Mayes LC Potenza MN Crowley MJ. Co-occurring tobacco and cannabis use in adolescents: dissociable relationships with mediofrontal electrocortical activity during reward feedback processing. Neuroimage Clin. (2021) 30:102592. 10.1016/j.nicl.2021.102592 33667977 PMC7932890

[77] Karoly HC Bryan AD Weiland BJ Mayer A Dodd A Feldstein Ewing SW. Does incentive-elicited nucleus accumbens activation differ by substance of abuse? An examination with adolescents. Dev Cogn Neurosci. (2015) 16:5–15. 10.1016/j.dcn.2015.05.005 26070843 PMC4657439

[78] Cabeen RP Allman JM Toga AW. THC exposure is reflected in the microstructure of the cerebral cortex and amygdala of young adults. Cereb Cortex. (2020) 30:4949–63. 10.1093/cercor/bhaa087 32377689 PMC7947183

[79] Gunasekera B Davies C Martin-Santos R Bhattacharyya S. The Yin and Yang of cannabis: a systematic review of human neuroimaging evidence of the differential effects of δ(9)-tetrahydrocannabinol and cannabidiol. Biol Psychiatry Cogn Neurosci Neuroimaging. (2021) 6:636–45. 10.1016/j.bpsc.2020.10.007 33414100

