# Supplementary material for: Age- and Sex-Related Cortical Gray Matter Volume Differences in Adolescent Cannabis Users: A Systematic Review and Meta-Analysis of Voxel-Based Morphometry Studies
Source: Front Psychiatry. 2021 Dec 1;12:745193. doi: 10.3389/fpsyt.2021.745193 (PMC8671465; doi:10.3389/fpsyt.2021.745193)
Supplement: Supplementary file 1 [file Data_Sheet_1.docx]

**Supplementary Online Content**

Allick A, Park G, Kim K, Vintimilla M, Rathod K, Lebo R, Nanavati J, Hammond CJ. Age- and sex-related cortical gray matter volume differences in adolescent cannabis users: A systematic review and meta-analysis of voxel-based morphometry studies. *Frontiers in Psychiatry*

**eMethods S1.** Additional methodologic details related to the meta-analytic procedure

**eResults S1.** Qualitative Analysis of Studies of GMV Differences Between Cannabis Using and Non-Using Typically Developing Youth

**eTable S1.** Study Characteristics of Studies of GMV Differences in the Qualitative Review

**eTable S2.** Controlling for covariates/confounders across studies included in the Meta-analysis

**eTable S3.** Age, Proportion of Females, and Other Variables used in Meta-regression analyses, Across studies

**eTable S4.** Results of Jack-knife Reliability Analyses of the Age-related Meta-regression

**eTable S5.**  Results of Jack-knife Reliability Analyses of the Sex-related Meta-regression

**eFigure S1**. Meta-regression Results Showing Associations between Years duration of Cannabis Use with Gray Matter Differences Between CU and TD youth

**eTable S6.**  Results of Jack-knife Reliability Analyses of the Years duration of cannabis use Meta-regression

**eMethods S1.** Additional methodologic details related to the meta-analytic procedure

Linear meta-regression analyses: Simple linear meta-regression analyses were carried out weighted by the square root of the sample size to predict SDM effect size values. The main output for each variable indicates the regression slope (i.e. amount of GMV change per unit increase in mean age, proportion of females, and duration of CU [mean years]). As our primary meta-analysis showed no significant differences between CU and TD youth, significant clusters from the age-related and sex-related meta-regression analyses can be interpreted as regions showing gray matter differences between CU and TD youth that varied as a function of age and the proportion of females in the datasets/studies.

**eResults S1.** Qualitative Analysis of Studies of GMV Differences Between Cannabis Using and Non-Using Typically Developing Youth

Our qualitative review is presented below with study characteristics shown in eTable S1.

**Search Results**: Using the search strategy, key words, and MesH terms presented in the methods section, we conducted searches in the online databases for PubMed/Medline, Cochrane, Embase, and Web of Science looking for citations published between January 1990 and November 2019. The initial search identified 2094 citations including 661 from PubMed/Medline, 884 from Embase, 439 from Web of Science, and 110 from Cochrane. After removal of 768 duplicates (36% overlap), 1327 unique citations were uploaded into Covidence software (covidence.org). These 1327 citations underwent title and abstract and subsequent full text reviews as a part of our systematic review. Of the initial 1327 citations, 822 records were excluded following title and abstract screen. Out of 436 citations that underwent full text review, 20 studies examining gray matter volume (GMV) differences between CU and TD youth^1-20^ were included in the qualitative analysis, 6 of which met all inclusion criteria. A PRISMA flow diagram depicting the search process is presented in Figure 1. Results from the qualitative analysis are presented below and in eTable S1.

**Qualitative analysis**: The 20 studies included in the qualitative analysis^1-20^ represented GMV data from 2,598 participants (approximately 50% CU youth). There was heterogeneity across study designs, analytic methods used, and sample characteristics. Many of the studies were underpowered, and a large number of studies used region-of-interest (ROI) analyses and small volume corrections (SVCs). A majority of the studies attempted to control for covariates/confounders in their analyses (see eTable 2 for covariates controlled for in the meta-analytic sample). Eighteen GMV studies reported group-level comparisons between CU and matched non-using TD youth and two GMV studies reported results from correlational analyses between GM volumes and cannabis use outcomes in samples of CU youth.

Group-level Comparisons between CU vs. TD youth: Focusing on the group-level comparisons (n=18 studies): seven studies (39% of the group-comparison subset sample) showed no GMV differences, and eleven studies (61% of the group-comparison subset sample) showed GMV differences between CU and non-using TD youth. Studies that reported GMV differences generally tended to have smaller sample sizes compared to studies that reported null findings.

*Global vs. Regional Effects of Adolescent Cannabis Exposure on GMV*: No global GMV differences were observed. All studies showing cannabis-related group-level GMV differences (n=11) reported regionally specific effects which were focal in some cases and more broadly distributed in others. Cortical and subcortical brain regions showing CU vs. TD GMV differences included the hippocampus, nucleus accumbens, caudate, putamen, striatum, amygdala, anterior insula, anterior cingulate cortex (ACC), middle and superior temporal gyri, PFC, medial and lateral orbital PFC (oPFC), precentral gyrus, inferior and superior parietal lobes, precuneus, superior and middle frontal gyri, middle occipital gyrus, fusiform gyrus, and cerebellum (anterior regions and vermis). These regions, particularly the subcortical and temporal regions, are notable for having elevated expression of CB1 receptors compared to other brain regions. This was investigated in one study: Orr et al., 2018 showed spatial coherence between their GMV results and voxel-wise expression of CB1 receptors from an independent PET imaging study in healthy adults.^1^

*Is Adolescent Cannabis Exposure Associated with Increased GM Volume, Decreased GM Volume or Neither?* In terms of directional relationships across the group-comparisons (n=18): seven studies (39%) reported increased GMV, four studies (22%) reported decreased GMV, and seven studies (39%) reported neither increased nor decreased GMV (i.e., null finding) between CU and matched non-using TD youth. Two studies reported both increased and decreased GMV in distinct regions.

Sex-by-Group Interaction Effects: Of the three studies that investigated sex-by-CU-group interaction effects, two reported significant sex differences in GMV between CU and TD youth^6,19^ and one reported a null finding.^2^ Both of the studies reporting sex-by-group-GMV effects noted increased GM volume in female CU and decreased or similar GM volumes in male CU compared to sex-matched controls. Brain regions showing sex-by-group GMV effects included the amygdala and PFC regions.

Correlational Analyses: Both of the studies that investigated relationships between GM volume and cannabis outcomes as their main *a priori* analysis, reported significant negative correlations between cannabis-related variables and GM volume. Specifically: Maple *et al.* 2019 found that lower rACC volume was associated with greater past year cannabis use^16^ and Padula *et al.,* 2015 found that lower amygdala volume was associated with greater severity of cannabis cravings during abstinence in CU youth.^7^ Secondary correlational analyses from other studies also identified significant relationships between GM volume and cannabis-related phenotypes and additionally identified associations between GM volume and mood/anxiety symptoms and cognitive functioning. Some of these associations varied as a function of sex (male vs. female) and group membership (CU vs. TD). Across studies, earlier age of onset and greater frequency, chronicity, and problems related to cannabis use were associated with lower GM volumes in CU youth.^3,5,12,13^ Mood and anxiety symptoms were linked to amygdala volume and showed sex-dependent associations - being negatively associated with amygdala volume in female controls and males and positively associated with amygdala volume in female CU.^11,19^ Associations between GM volume and cognitive function were more complex, with some studies showing opposite brain-cognition relationships in CU and TD youth.^4,6,16^ For example, in two studies that examined relationships between GMV and executive function (EF) test scores, smaller PFC and cerebellar volumes were associated with better EF test scores in CU youth and the opposite pattern was seen in non-using TD youth.^4,6^ In contrast, Maple et al., 2019 found that smaller left rACC volumes were associated with lower scores on a test of visuospatial memory in both CU and TD youth.^16^

**eTable S1.** Study Characteristics of Studies of GMV Differences in the Qualitative Analysis

| Source | Type of Participants, No. Used in Analysis) | Age, Mean (SD) | Male Sex No. (%) | Addiction Diagnosis and Severity | Abstinence on scan day | Single vs. Multiple Scans | MRI scanner, power | Scan Parameters | Analysis | Results |
| --- | --- | --- | --- | --- | --- | --- | --- | --- | --- | --- |
| Ashtari et al., 2011 | N= 14 Males Heavy Cannabis Users N=14 Healthy Controls | Heavy MJ Users= 19.3(0.8) Healthy Controls=18.5(1.4) | Cannabis Users = 100% Healthy Control= 100% | Cannabis users DSM-IV diagnostic criteria for cannabis dependence in remission, excluded if they had more than five-lifetime exposures with illicit drugs, tobacco, and alcohol | In-patient supervision with monitored toxicology, people, abstained for approximately six months | Single Scan | 1.5T Ge Neuro Vascular Interactive (NV/i) | TR/TE 10.1/4.2 ms; bandwidth = 21 kHz, matrix size = 256×192, FOV = 22×22 cm2, inversion time = 600 ms, NEX = 1) | ROI-based analysis of VBM data focused on hippocampus and amygdala brain regions. | Group-level analysis Heavy-cannabis users showed significantly smaller R/L hippocampal volumes, but no significant differences in the amygdala volume compared to controls. Correlation analysis In cannabis users, a smaller right hippocampus volume was correlated with a higher amount of cannabis use |
| Cousijn et al. 2012 | N= 33 Heavy Cannabis Users N=43 Controls | Cannabis Users= 21.3(2.4) Controls=21.9(2.4) | Cannabis Users= 64% Controls= 62% | Users had an average CUDIT score 12.4(5.7) | None | Single Scan | 3T MRI scanner (Phillips Intera, Best, The Netherlands) | TR/TE=9.6s/4.6ms, 182 slices, slice thickness=1.2mm FOV=256*256mm, in-plane resolution=256*256, FA=8° | Whole-brain voxel-wise analysis using VBM. Grey matter volume of the entire brain was calculated. | Group-level analysis Heavy cannabis using adolescents had larger L/R anterior cerebellum volumes compared to Controls, but did not differ from controls in volumes of other brain regions. Correlation analysis Among heavy CU adolescents, amygdala and hippocampal volumes correlated negatively with the amount of cannabis use or problem-severity scores. |
| Churchwell et al. 2010 | N= 18 Cannabis Abusers N= 18 Healthy Controls | Cannabis Abusers= 17.7(.94) Healthy Controls=17.7(.82) | Cannabis Users= 88.7% Controls = 66.7% | All cannabis users met the criteria for DSM-IV cannabis abuse or dependence | None | Single Scan | 3T Trio Scanner | TR/TE=2000/3.38ms FOV=256mm FA=8, 1mm slice thickness | VBM ROI analysis focused on medial orbitofrontal PFC region (moPFC) and lateral PFC region | Group-level analysis Cannabis abusing adolescents had decreased moPFC volumes and greater self-reported impulsivity scores compared to Non-Using Healthy Controls. Correlation analysis Total mOPFC volume was positively correlated with age of onset. |
| Churchwell et al., 2012 | N=27 subjects including N=10 healthy controls, N=9 Methamphetamine Users and N=8 Meth + MJ co-users | HC:  Meth Users:  Meth+MJ co-users: | HC:  Meth Users:  Meth+MJ co-users: | KSADS-PL interview: All subjects with Meth Use met DSM-IV criteria for Methamphetamine Abuse/Dep and all subjects with Meth+MJ co-use met criteria for both Meth and Cannabis Abuse/Dep | Not Reported | Single scan | 3T Siemens Trio scanner | TE/TR: 3.38/3.0 ms, FA=8, FOV = 256 mm, 1 mm slice thickness | Voxel-wise ROI analysis of GM volume focused on striatal ROI including caudate, accumbens, and putamen | Group-level analysis Meth+MJ users had decreased left putamen volume compared to HC and Meth Users who did not differ in GMV. |
| Gilman et al. 2014 | N=20 Cannabis Users N=20 Healthy Controls | Cannabis Users=21.3(1.9) Controls=20.7(1.9) | Cannabis Users = 43% Healthy Control= 43% | Used marijuana at least once a week but did not qualify for the DSM-IV criteria for cannabis dependence | None asked to abstain the day of the scan | Single Scan | 3T Siemens Trio scanner | FOV 256 mm, TR/TI= 2530/1200 ms, 2X GRAPPA acceleration, TE 1.64/3.5/5.36/7.22 ms, BW 651 Hz/px, Functional scans= 2D gradient echo EPI sequence (31 slices, 3 mm thick, 0.6mmgap, FOV216mm,3mm2 in-plane resolution, TR/TE=2s/30ms, BW 2240 Hz/px) | Whole-brain voxel-wise analysis of grey matter density and volume using VBM. | Group-level analysis For GMV: MJ users had increased nucleus accumbens volumes compared to HC that reached trend-level.  For GM density: MJ users had increased GM density in the left nucleus accumbens extending into the subcallosal cortex, hypothalamus, amygdala, and SL-extended amygdala after controlling for age, sex, alcohol use, and cigarette smoking. |
| Jarvis et al., 2008 | N=14 participants including N=7 with bipolar disorder (BP) and comorbid cannabis use disorder (CUD) and N=7 with BP w/o CUD | BP w/ CUD: 15.0  BP w/o CUD: 16.0 | BP w/ CUD: 29%  BP w/o CUD: 43% | SCID DSM-IV, ASI were used to obtain CUD diagnosis | All BP w/ CUD participants were > 72 hours abstinent at time of scan (based upon inpatient setting) | Single scan | 3T Burker Biospec scanner | TR/TE=16.5/4.3 ms, TI=550ms, FOV=25.6x19.2x14.4, FA=20 | whole-brain voxel-based VBM analysis comparing GMV and GM density between groups | Group-level analyses: BP w/ CUD patients had decreased GMV in left fusiform gyrus and increased GMV in the right caudate and precentral gyrus and increased GM density in the right middle occipital gyrus, right fusiform gyrus, and cerebellar vermis compared to BP w/o CUD patients. |
| Kumra et al. 2012 | N= 35 Early-Onset Schizophrenia (EOS) N= 51 Healthy Controls N= 16 Cannabis Use Disorder (CUD) N=13 Early Onset Schizophrenia + Cannabis Use Disorder (EOS+ CUD) | Healthy Controls= 16.2(2.3) CUD=16.6(1.7) EOS=16.5(1.8) EOS+CUD=15.7(2) | Healthy Controls=49% CUD=93.8% EOS=82.9% EOS+CUD=84.6% | by age of 17 they had more than 50 lifetime uses of marijuana in their lifetime. | Recruited from treatment centers so their last days of use was between 15-648 days. | Single Scan | Siemens 3.0T MR System | TE/TR/TI=3.65ms/2,530ms/1,100ms FA=7°, FOV=256*176mm, number slices= 224 FLASH TR/TE=18/4.3ms FA=25°, FOV= 240mm, in-plane resolution=0.625mm*0.62mm, slice thickness=1.5mm, NEX=1 | VBM ROI analysis focused on the following a priori regions: left and right superior parietal cortex, superior frontal, middle frontal, inferior frontal, lateral orbitofrontal, and medial orbitofrontal gyri, parietal (inferior parietal lobule, post-central gyrus, supramarginal gyrus, precuneus), temporal (superior temporal gyri), limbic (hippocampus, anterior cingulate) lobes, and subcortical regions (caudate, thalami). | Group-level analyses A significant EOS-by-CUD interaction was observed. In the left superior parietal region, EOS and CUD-only groups both had smaller GMV compared to HC.  Focusing on CU/CUD effects: In the left superior parietal as well as the left medial orbitofrontal, left lateral orbitofrontal, left rostral anterior cingulate, right and left rostral middle frontal, and right superior frontal lobes CUD status had directionally opposite relationship w/ brain morphometry in youth with EOS compared to HC. For example, for most brain regions CUD status in relation to EOS (i.e. comparing CUD+EOS vs. EOS-only) was associated with larger brain volumes whereas CUD status was associated with smaller brain volumes in relation to HC. There were 2 exceptions: First, in the right and left hippocampus, in HC, there was no distinguishable effect of a CUD, whereas the effect of CUD in adolescents with schizophrenia was associated with larger volumes. Second, in the left thalamus, the effect of CUD in healthy controls was associated with larger brain volumes, whereas the effect of CUD in adolescents with schizophrenia was associated with smaller brain volumes. |
| Maple et al. 2019 | N= 20 Cannabis Using N=35 Controls | Cannabis Female= 21.4(2.0) Controls Females=20.8(2.5) Cannabis Males=22.4(2.4) Controls Males=20.5(3.2) | MJ Users= 60% Controls=45.7% | Cannabis users were required to have at least 40 uses in the past year and 50 lifetime uses. | Three weeks of monitored abstinence. | Single Scan | 3T Scanner (GE Healthcare) | TR/TE/TI=8.2ms/3.4s/450 FA=12°, In-plane resolution-256*25 FOV=240mm Slice thickness-1mm 150 slices | VBM ROI analysis examining relationship between cognitive scores, GMV, and cananbis use variables. Regression analysis was used to carry out the relationship between past-year cannabis use, cannabis x gender interaction, and brain structure using the following region's volumes as dependent variables (pars triangularis, superior temporal, fusiform, rostral ACC, caudal ACC, amygdala, hippocampus, cerebellum | Correlation analysis In the combined group including both cannabis users and non-users, greater past year use of cannabis was significantly correlated with smaller left rACC volumes. In addition, smaller rACC volumes were associated with lower Discrimination Correct Scores.  Group-level analysis: No group-level comparisons were made between CU and Controls in this study. |
| McQueeny et al. 2011 | N= 35 Chronic Marijuana Users N=47 Healthy Controls | Female Users= 18.15(.86) Male Users=17.92(.91) | Marijuana Chronic Users=77% Healthy Controls=77% | Marijuana users had been using marijuana for at least three years 50% of the female users met the criteria for cannabis dependence, and 81% | Asked to abstain for 28 weeks before scan, monitored abstinence was used to assure compliance | Single Scan | 3-Telsa General Electric Scanner | 3D TI (TR/TE=8/3ms FA=12⸰ FOV= 240mm, 176 continuous slices, slice thickness=1mm, in-plane resolution=1*1mm | VBM ROI analysis focused on the amygdala as a ROI. The volumes of the amygdala were calculated, and regression analysis was run to determine the correlations between amygdala volumes and mood and anxiety symptoms | Group-level analysis MJ users did not differ from controls in amygdala volume, but a group-by-gender interaction was observed. Female MJ users had larger right amygdala volumes and more internalizing symptoms than female CON, while male MJ users had similar volumes as male controls. Correlation analysis For female CON and males, worse mood/anxiety was linked to smaller right amygdala volume, whereas more mood/anxiety symptoms was associated with bigger R amgydala volume in female MJ users. |
| Medina et al. 2009 | N=16 MJ Users, N=16 Healthy Controls | Male Controls=17.7(1.1) Female Controls= 18.2(0.6) Male MJ Users = 18.1(0.8) Female MJ Users= 18.2(0.6) | MJ Users= 75% Healthy Controls= 63% | MJ users had Lifetime MJ 60 uses, past month marijuana use, | monitored abstinence for 28 days with a urine test and Breathalyzer tests | Single Scan | 1.5 Tesla GE Signa LX system | TR/TE=2000/16ms FOV=240mm, Voxel Dimensions=0.9375*.9375*1.328, 128 continuous slices | VBM ROI volumetric analysis focused on PFC and PFC subregion volumes (total, anterior dorsal, anterior ventral, and posterior) and on white matter volume (WMV) total (whole-brain) and in PFC and PFC subregions. | Group-level analysis MJ users did not differ from controls in PFC volume, but a group-by-gender interaction was observed whereby female MJ users had comparatively larger PFC volumes and male MJ users had comparatively smaller PFC volumes compared to sex-matched controls.  Correlation analysis Group status and PFC volume interacted to predict executive function. Among MJ users, smaller PFC volumes were associated with better EF test scores while the opposite pattern was observed in controls. |
| Medina et al. 2010 | N=16 MJ Users, N=16 Healthy Controls | MJ users = 18.1 (0.7); Controls = 18.0 (1.0) | MJ users = 75%  Healthy Controls = 63% | MJ users had Lifetime MJ 60 uses, past month marijuana use, | MJ users had Lifetime MJ 60 uses, past month marijuana use, | Single Scan | 1.5 Tesla GE Signa LX system | TR/TE=2000/16ms FOV=240mm, Voxel Dimensions=0.9375*.9375*1.328, 128 continuous slices | VBM ROI-based volumetric analysis of GMV focused on cerebellum and cerebellar regions | Group-level analysis Adolescent MJ users demonstrated significantly larger inferior posterior (lobules VIII–X) vermis volume than controls, above and beyond effects of lifetime alcohol and other drug use, sex, and ICV.  Correlation analysis Larger vermis volumes were associated with poorer executive functioning.  Following 1 month of abstinence, adolescent MJ users had significantly larger posterior cerebellar vermis volumes than controls. |
| Medina et al. 2007 | N= 21 Healthy Control N=16 Alcohol N=26 MJ+ALC | Healthy Controls= 17.5(1.1) Alcohol=16.9(0.7) MJ+ALC= 17.6(0.9) | Healthy Controls=65% Alcohol=77% MJ+ALC=73% | MJ+ALC users had more than 40 lifetimes uses of MJ and more than 60 uses of alcohol | Required two days of abstinence | Single Scan | 1.5 Tesla GE Signa LX system | TR/TE=2000/16ms FOV=240mm, Voxel Dimensions=0.9375*.9375*1.328, 128 continuous slices | VBM ROI GMV analysis focused on b/l hippocampus | Group-level analysis ALC users had reduced left hippocampal volume compared to MJ+ALC users and controls who did not differ from each other on GMV outcomes. Correlation analysis Increased alcohol use problem severity was associated with increased  right > left ( R>L) asymmetry and smaller left hippocampal volumes while marijuana abuse/dependence was associated with increased L>R  asymmetry and larger left hippocampal volumes. |
| Medina et al. 2007 | N=16 MJ Users, N=16 Healthy Controls | Healthy Controls=18(0.9) MJ Users=18(0.7) | MJ Users= 75% Healthy Controls=69% | MJ users had at least 60 lifetimes uses | Monitored abstinence for 28 days | Single Scan | 1.5 Tesla General Electric SignaLX | TR/TE=2000/16ms FOV=240mm, Voxel Dimensions=0.9375*.9375*1.328, 128 continuous slices | VBM ROI GMV analysis focused on b/l hippocampal volumes (hippocampal: ICV ratio) and whole-brain white matter volume (WMV) analysis | Group-level analysis No group-level differences between MJ users and HC in hippocampal volume and white matter volume. Correlation analysis WMV was predictive of depressive symptoms in MJ users. |
| Orr et al. 2019 | Cohort 1: N= 46 MJ Users Controls=46 Cohort 2: N=69 Cannabis Users Healthy Controls= 69 | Cohort 1: Cannabis Users=14.60 Controls= 14.51 Cohort 2: Cannabis=14.43 Controls= 14.50 | Cohort 1: Cannabis Users= 65% Controls= 48% Control 2: Cannabis=74% Controls=70% | Cohort 1: 1 or 2 lifetime uses Cohort 2: 10 more instances | No treatment | Single Scan | 3T whole-body MRI systems | Protocol from ADNI | Whole-brain voxel wise analyses using VBM were conducted, and grey matter volume was calculated | Group-level analysis Low-level early-adolescent cannabis users had larger volumes in a number of brain regions compared to non-using age-matched controls. Low levels of cannabis use in cohort one was associated with greater grey matter volume in the hippocampus, amygdala, and striatum, bilateral parietal regions, cerebellum, and left middle temporal gyrus. Correlation analysis In addition, the magnitude of differences in GMV were associated with CB1 receptor availability from a separate dataset. |
| Padula et al. 2015 | N=22 Cannabis Users | Cannabis Users= 17.8(0.9) | Cannabis Users=77% | Cannabis users used 200 or more lifetime uses of marijuana | Abstinent for at least 28 days | Single Scan | 3T GE scanner | TR/TE=8/3ms FA=12°, FOV= 240mm,176 continuous slices, 1 mm3, | VBM ROI analysis focused on amygdala volume and correlating amygdala volume with cannabis use variables in MJ users | Correlation analysis In adolescent MJ users during early-abstinence (7-days), smaller amygdala volumes were associated w/ higher cannabis cravings scores.  Group-level analysis This study had no control group and thus no group-level comparisons between MJ and CONs. |
| Price-Lisdahl et al. 2015 | N=27 Cannabis Users N=32 Controls | MJ Users= 21.41(2.21) Controls= 21.09(2.32) | MJ Users= 55.56% Healthy Controls: 43.7% | At least 25 uses in the past year and more than 50 uses in a lifetime | None | Single Scan | 4T Varian MRI scanner | TMD/TR/TE=1.1s/13ms/6ms FOV=25.6*19.2*19.2, Matrix 256*192*96 pixels FA=20° | VBM ROI analysis focused on the following ROIs: lateral orbitofrontal cortex, medial orbitofrontal cortex (moPFC), superior frontal, rostral middle frontal, and inferior parietal cortex were measured | Group-level analysis MJ users had significantly smaller moPFC cortex when compared to non-using controls; Correlation analysis smaller moPFC cortex volumes were linked to worse scores on the Paced Auditory Serial Attention Test in MJ users. |
| Scott et al. 2019 | N= 634 Non-Users, N= 109 Occasional cannabis users, N=38 Frequent cannabis users | Non-Users=17.0(2.1) Occasional cannabis users=18.1(2.0) Frequent cannabis users=18.5(1.6) | Non-Users=40% Occasional cannabis users=50% Frequent cannabis users=71% | Frequent users used cannabis 3 or 4 times or week, while occasional users used it one to two times a week. | None | Single Scan | 3T Siemens Trio Scanner | TR/TE=1810/3.51ms FOV=180*240mm Matrix=192*256, 160 slices, slice thickness/gap 1/0mm, TI=1100ms FA=9°, voxel size= .93*.93*1.00mm total acquisition=3:28min | Cortical thickness, volume, and grey matter density were measured | Group-level analysis A number of brain regions showed CU vs. Non-User group-level differences across measures of cortical thickness, volume, GMD, in uncorrected analyses, but no differences across CU vs. Non-Use groups were significant after completing Kruskall–Wallis tests and FDR for statistical correction. No age- or sex-related effects were identified in post-hoc analyses. One pair-wise difference between CU and Non-Users remained significant after FDR correction: CU had reduced cortical thickness in left frontal lobe compared to Non-Users. |
| Sultan et al. 2021 | N=144 adolescents total including: N=44 BP w/ CU, N= 34 BP w/o CU, N=63 HC w/o CU | BP w/ CU: 17.45 (1.18)  BP w/o CU: 17.07 (1.70)  HC w/o CU: 17.00(1.68) | BP w/ CU:34%  BP w/o CU:41%  HC w/o CU:46% | CU assessed via self-report response to K-SADS supplement. All BP w/ CU had past year CU and 13/44 met DSM-5 criteria for CUD | None | Single scan | 3T Phillips Achieva Scanner | TR/TE=9.5/2.3ms, IT=1400ms, SR=0.94x1.17x1.2mm2, 256x164x140 matrix, FA=8, FOV= 240x191 mm2, scan duration = 8m56s, 140 slices | Cortical thickness, surface area, volume | Group-level analysis In vertex-wise analyses, group-level differences were observed in several frontal and parietal regions. Compared to HC, BP youth w/ CU had larger volume and surface area in parietal regions (Inferior parietal, superior parietal lobe) and precuneus and reduced thickness in frontal regions (precentral gyrus, medial OFC). Compared to HC, BP w/o CU had reduced volume, surface area, and thickness in parietal and frontal regions.  ROI analyses revealed no group-level differences. |
| Thayer et al. 2017 | Adult Cannabis Users= 191 Adult Nonusing Controls= 662 ; Adolescents Cannabis Users=201 Adolescent Nonusing Controls=238 | Adults (whole sample):31.34(9.64) Adults(cannabis users): 28.81(8.44) Adolescent(whole sample)=15.97(1.17) Adolescents(cannabis users)=16.00(1.08) | Adults (whole sample):62% Adults(cannabis users): 69% Adolescent(whole sample)=69% Adolescents(cannabis users)=74% | Used marijuana weekly | None | Single Scan | 3T Siemens Trio Scanner | TE=1.64, 3.50,5.36,7.22, and 9.08ms. TR=2.53s TI=1.20s FA= 7° NEX=1 slice thickness=1mm, 192 saggital slices, FOV=256*256mm resolutions 256*256*176 voxel size=1*1*1mm Pixels Banswidth=650Hz DTI FOV=256*256mmMatrix=128*128, slice thickness=2mm NEX=1 TE=84ms TR=9000ms | Whole-Brain Voxel-wise GMV analysis examining CU vs. TD differences | Group-level analysis No group-level differences in GMV were observed between CU and TD youth. |
| Weiland et al. 2015 | Adult MJ Users=29 Adult Nonusers=29 Adolescent Users=50 Adolescent Nonusers=50 | Adult nonusers=27.5(6.8) Adult daily users=27.4(7.1) Adolescents Nonusers=16.77(.95) Adolescents Daily Users=16.65(1.09) | Adult nonusers=55.2% Adult daily users=55.2%Adolescents Nonusers=72% Adolescents Daily Users=82% | In the past 90 days both adolescents and adults used marijuana daily | None | Single Scan | 3T Siemens Trio Scanner | TE=1.64, 3.50,5.36,7.22, and 9.08ms. TR=2.53s TI=1.20s FA= 7° NEX=1 slice thickness=1mm, FOV=256mm resolutions 256*256*176 voxel size=1*1*1mm Pixels Bandwidth=650Hz DTI FOV=256*256mmMatrix=128*128, slice thickness=2mm NEX=1 TE=8 | Whole brain voxel-wise VBM analysis as well as ROI analysis bilateral nucleus accumbens, amygdala, hippocampi, and the cerebellum were performed | Group-level analysis There was no significant difference in any brain region between cannabis users and controls |

**eTable S2.** Controlling for covariates/confounders across studies included in the Meta-analysis

| **Studies** | Attempted to Control for alcohol use | Attempted to  Control for  tobacco use | Excluded youth with comorbid psychiatric disorders |
| --- | --- | --- | --- |
| Gilman et al. | Yes | Yes | Yes |
| Thayer et al. | Yes | No | No |
| Weiland et al. | Yes^a^ | No | No |
| Orr et al. | Yes^a^ | Yes | Yes |
| Cousijn et al. | Yes | Yes | Yes |
| Jarvis et al. | No | No | No^b^ |

**Note**: a= Weiland et al. and Orr et al. matched participants and controls on alcohol use; b= Jarvis et al. included youth with bipolar disorder with and without comorbid cannabis use disorder.

**eTable S3.** Age, Proportion of Females, and Other Variables used in Meta-regression analyses, Across studies

| **Studies** | Mean Age CU youth  (years) | Proportion of Female participants (%) | Age range (years) | Average Past 30-Day CU (days) | Duration of CU  (years) |
| --- | --- | --- | --- | --- | --- |
| Gilman et al. | 21.3 | 55% | 7 | 15.3 | 6.2 |
| Thayer et al. | 15.97 | 31% | 4 | 9.7 | NR |
| Weiland et al. | 16.65 | 18% | 4 | 30.0 | 3.4 |
| Orr et al. | 14.6 | 35% | 1 | NA | 0.004 |
| Cousijn et al. | 21.3 | 36% | 7 | 19.3 | 2.5 |
| Jarvis et al. | 15 | 71% | 6 | 0 | NR |

**Note**: NA = Not applicable; NR = not reported/provided

**eTable S4.** Results of Jack-knife Reliability Analyses of the Age-related Meta-regression analysis

| **Studies** | **Left superior temporal gyrus**  **(-52,-6,-12)** |
| --- | --- |
| Gilman et al. | No |
| Thayer et al. | Yes |
| Weiland et al. | Yes* (-56,-6,-10) |
| Orr et al. | No |
| Cousijn et al. | Yes* (-50,-6,-12) |
| Jarvis et al. | Yes |

**Note**: Yes – brain region remains significantly decreased in CU vs. TD youth as a function of age following exclusion of this study/dataset as part of the jackknife sensitivity analysis; No – brain region is no longer significantly decreased as a function of age when the study/dataset is removed; *- denotes that the left superior temporal gyrus finding remained significant in meta-regression analyses when this dataset/study was removed but the peak of the foci was located at slightly different coordinates

**eTable S5.**  Results of Jack-knife Reliability Analyses of the Sex-related Meta-regression analysis

| **Studies** | **Right middle occipital gyrus (BA19)**  **(36,-80,28)** |
| --- | --- |
| Gilman et al. | Yes* (38,-86,26) |
| Thayer et al. | Yes |
| Weiland et al. | Yes |
| Orr et al. | Yes |
| Cousijn et al. | Yes |
| Jarvis et al. | No |

**Note**: Yes – brain region remains significantly increased in CU vs. TD youth as a function of proportion of females following exclusion of this study/dataset as part of the jackknife sensitivity analysis; No – brain region is no longer significantly increased as a function of proportion of females when the study/dataset is removed; *- denotes that the right middle occipital gyrus finding remained significant in meta-regression analyses when this dataset/study was removed but the peak of the foci was located at slightly different coordinates


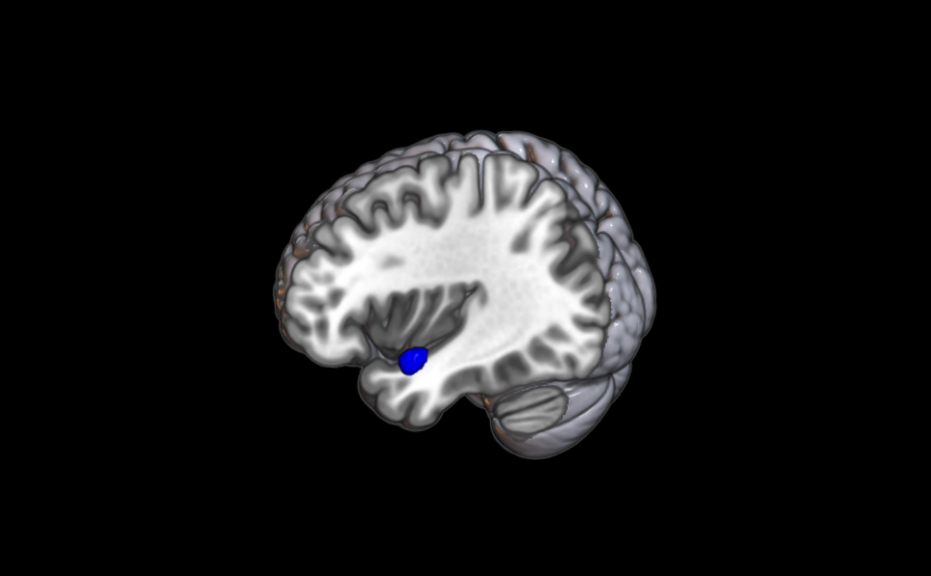

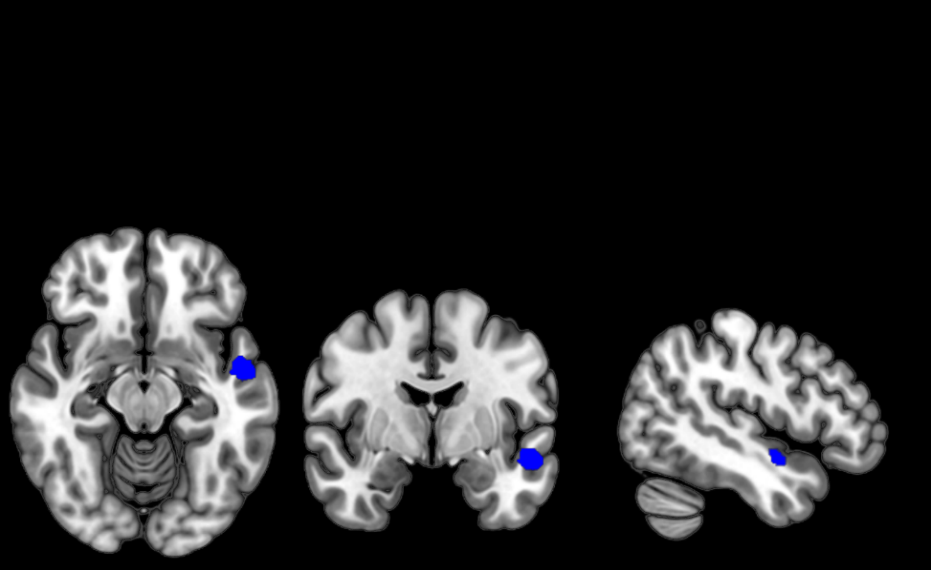
**eFigure S1**. Meta-regression Results Showing Associations between Years duration of Cannabis Use with Gray Matter Differences Between Cannabis Using and Typically Developing Youth

**A...**

**B.**

Years Duration of Cannabis Use meta-regression results. **A.** Meta-regression results (CU > TD youth) showing associations between duration of cannabis use in years and gray matter differences between CU and TD youth shown in blue. All results thresholded at p < 0.005. **B.** Associations between years duration of CU and gray matter differences in the left superior temporal cortex (145 voxels, SDM-Z = -3.542) (shown in blue). Effect sizes (SDM-estimates) used to create the meta-regression plots were extracted from the peak of maximum slope significance. The meta-regression SDM-estimate value is derived from the proportion of studies that reported gray matter changes near the voxel so it is expected that some values are at 0 or near +/- 1. Each included study is represented as a numbered dot, with the dot size reflecting relative total sample size of each specific study in comparison to the average total sample size of all six studies included in the regression. Study key: 1 = Gilman et al. 2014; 2 = Thayer et al., 2017; 3 = Weiland et al., 2015; 4 = Orr et al., 2019; 5 = Cousijn et al. 2012; 6 = Jarvis et al., 2008.

**eTable S6.**  Results of Jack-knife Reliability Analyses of the Duration of Cannabis Use Meta-regression analysis

| **Studies** | **Left superior temporal gyrus (BA21)**  **(52,-4,-14)** |
| --- | --- |
| Gilman et al. | No |
| Thayer et al. | Yes |
| Weiland et al. | Yes |
| Orr et al. | No |
| Cousijn et al. | Yes*(52,-2,-14) |
| Jarvis et al. | Yes |

**Note**: Yes – brain region remains significantly decreased in CU vs. TD youth as a function of duration of cannabis use (years) following exclusion of this study/dataset as part of the jackknife sensitivity analysis; No – brain region is no longer significantly decreased as a function of duration of CU when the study/dataset is removed; *- denotes that the left superior temporal gyrus finding remained significant in meta-regression analyses when this dataset/study was removed but the peak of the foci was located at slightly different coordinates

**Supplemental Online Material References:**

1. Orr C, Spechler P, Cao Z, et al. Grey Matter Volume Differences Associated with Extremely Low Levels of Cannabis Use in Adolescence. *J Neurosci.* 2019;39(10):1817-1827.

2. Scott JC, Rosen AFG, Moore TM, et al. Cannabis use in youth is associated with limited alterations in brain structure. *Neuropsychopharmacology.* 2019;44(8):1362-1369.

3. Ashtari M, Avants B, Cyckowski L, et al. Medial temporal structures and memory functions in adolescents with heavy cannabis use. *J Psychiatr Res.* 2011;45(8):1055-1066.

4. Medina KL, Nagel BJ, Tapert SF. Abnormal cerebellar morphometry in abstinent adolescent marijuana users. *Psychiatry Res.* 2010;182(2):152-159.

5. Medina KL, Schweinsburg AD, Cohen-Zion M, Nagel BJ, Tapert SF. Effects of alcohol and combined marijuana and alcohol use during adolescence on hippocampal volume and asymmetry. *Neurotoxicology and teratology.* 2007;29(1):141-152.

6. Medina KL, McQueeny T, Nagel BJ, Hanson KL, Yang TT, Tapert SF. Prefrontal cortex morphometry in abstinent adolescent marijuana users: subtle gender effects. *Addict Biol.* 2009;14(4):457-468.

7. Padula CB, McQueeny T, Lisdahl KM, Price JS, Tapert SF. Craving is associated with amygdala volumes in adolescent marijuana users during abstinence. *Am J Drug Alcohol Abuse.* 2015;41(2):127-132.

8. Weiland BJ, Thayer RE, Depue BE, Sabbineni A, Bryan AD, Hutchison KE. Daily marijuana use is not associated with brain morphometric measures in adolescents or adults. *J Neurosci.* 2015;35(4):1505-1512.

9. Thayer RE, YorkWilliams S, Karoly HC, et al. Structural neuroimaging correlates of alcohol and cannabis use in adolescents and adults. *Addiction.* 2017;112(12):2144-2154.

10. Jarvis K, DelBello MP, Mills N, Elman I, Strakowski SM, Adler CM. Neuroanatomic comparison of bipolar adolescents with and without cannabis use disorders. *J Child Adolesc Psychopharmacol.* 2008;18(6):557-563.

11. Medina KL, Nagel BJ, Park A, McQueeny T, Tapert SF. Depressive symptoms in adolescents: associations with white matter volume and marijuana use. *J Child Psychol Psychiatry.* 2007;48(6):592-600.

12. Cousijn J, Wiers RW, Ridderinkhof KR, van den Brink W, Veltman DJ, Goudriaan AE. Grey matter alterations associated with cannabis use: results of a VBM study in heavy cannabis users and healthy controls. *Neuroimage.* 2012;59(4):3845-3851.

13. Churchwell JC, Lopez-Larson M, Yurgelun-Todd DA. Altered frontal cortical volume and decision making in adolescent cannabis users. *Front Psychol.* 2010;1:225.

14. Gilman JM, Kuster JK, Lee S, et al. Cannabis Use Is Quantitatively Associated with Nucleus Accumbens and Amygdala Abnormalities in Young Adult Recreational Users. *Journal of Neuroscience.* 2014;34(16):5529-5538.

15. Kumra S, Robinson P, Tambyraja R, et al. Parietal lobe volume deficits in adolescents with schizophrenia and adolescents with cannabis use disorders. *Journal of the American Academy of Child and Adolescent Psychiatry.* 2012;51(2):171-180.

16. Maple KE, Thomas AM, Kangiser MM, Lisdahl KM. Anterior cingulate volume reductions in abstinent adolescent and young adult cannabis users: Association with affective processing deficits. *Psychiatry Research: Neuroimaging.* 2019;288:51-59.

17. Price JS, McQueeny T, Shollenbarger S, Browning EL, Wieser J, Lisdahl KM. Effects of marijuana use on prefrontal and parietal volumes and cognition in emerging adults. *Psychopharmacology (Berl).* 2015;232(16):2939-2950.

18. Sultan AA, Kennedy KG, Fiksenbaum L, MacIntosh BJ, Goldstein BI. Neurostructural Correlates of Cannabis Use in Adolescent Bipolar Disorder. *The international journal of neuropsychopharmacology.* 2021;24(3):181-190.

19. McQueeny T, Padula CB, Price J, Medina KL, Logan P, Tapert SF. Gender effects on amygdala morphometry in adolescent marijuana users. *Behav Brain Res.* 2011;224(1):128-134.

20. Churchwell JC, Carey PD, Ferrett HL, Stein DJ, Yurgelun-Todd DA. Abnormal striatal circuitry and intensified novelty seeking among adolescents who abuse methamphetamine and cannabis. *Dev Neurosci.* 2012;34(4):310-317.
